# Variations in radioactive cesium accumulation in wheat germplasm from fields affected by the 2011 Fukushima nuclear power plant accident

**DOI:** 10.1038/s41598-020-60716-w

**Published:** 2020-02-28

**Authors:** Katashi Kubo, Hiroyuki Kobayashi, Miyuki Nitta, Shotaro Takenaka, Shuhei Nasuda, Shigeto Fujimura, Kyoko Takagi, Osamu Nagata, Takeshi Ota, Takuro Shinano

**Affiliations:** 1 Agricultural Radiation Research Center, Tohoku Agricultural Research Center, National Agriculture and Food Research Organization (NARO), 50 Harajukuminami, Arai, Fukushima 960-2156 Japan; 2 Biodiversity Division, Institute for Agro-Environmental Sciences, NARO, 3-1-3 Kannondai, Tsukuba, Ibaraki 305-8604 Japan; 3 Laboratory of Plant Genetics, Graduate School of Agriculture, Kyoto University, Kitashirakawaoiwake-cho, Sakyo-ku, Kyoto 606-8502 Japan; 4 Faculty of Agriculture, Ryukoku University, 1-5 Yokotani, Setaoe-cho, Otsu, Shiga 520-2194 Japan; 5 Bio-oriented Technology Research Advancement Institution, NARO, 8 Higashida-cho, Kawasaki, Kanagawa 210-0005 Japan; 6 Laboratory of Plant Nutrition, Research Faculty of Agriculture, Hokkaido University, Kita 9 Nishi 9, Kita-ku, Sapporo, Hokkaido 060-8589 Japan

**Keywords:** Natural variation in plants, Element cycles

## Abstract

Decreasing the transfer of radioactive cesium (RCs) from soil to crops has been important since the deposition of RCs in agricultural soil owing to the Fukushima nuclear power plant accident of 2011. We investigated the genotypic variation in RCs accumulation in 234 and 198 hexaploid wheat (*Triticum* spp.) varieties in an affected field in 2012 and 2013, respectively. The effects of soil exchangeable potassium (ExK) content to RCs accumulation in wheat varieties were also evaluated. A test field showed fourfold differences in soil ExK contents based on location, and the wheat varieties grown in areas with lower soil ExK contents tended to have higher grain RCs concentrations. RCs concentrations of shoots, when corrected by the soil ExK content, were positively significantly correlated between years, and RCs concentrations of shoots were significantly correlated with the grain RCs concentration corrected by the soil ExK content. These results indicated that there were genotypic variations in RCs accumulation. The grain to shoot ratio of RCs also showed significant genotypic variation. Wheat varieties with low RCs accumulations were identified. They could contribute to the research and breeding of low RCs accumulating wheat and to agricultural production in the area affected by RCs deposition.

## Introduction

Radioactive cesium (RCs) isotopes ^134^Cs and ^137^Cs, which can be incorporated into the food chain, becoming a threat to human health^1^, are two of the major radionuclides released by the accident at the Tokyo Electric Power Company’s Fukushima Dai-ichi (No. 1) nuclear power plant (TEPCO’s FDNPP), caused by the Great East Japan Earthquake and subsequent tsunami on 11 March 2011^2^. They dispersed into the environment, including agricultural land in eastern Japan, leading to concerns regarding the contamination of food with radioactive materials and their effects on human health. The Japanese Ministry of Health, Labor and Welfare established a provisional maximum regulatory value for RCs in agricultural products at 500 Bq kg^−1^on 17 March 2011. This was changed to 100 Bq kg^−1^ on 1 April 2012^3^. Although incidents of RCs contamination greater than the regulatory value in agricultural products have been limited^4^, the radioactivity monitoring of agricultural products has continued in the affected areas because of the relatively long half-life (30.2 years) of ^137^Cs. Studies and countermeasures to decrease RCs in agricultural products have been important in allowing the resumption of agricultural practices in the areas affected by RCs.

For wheat (*Triticum aestivum* L.) and barley *(Hordeum vulgare* L.), 27 of 557 samples collected from eastern Japan exceeded 100 Bq kg^−1^, with 1 exceeding 500 Bq kg^−1^, in 2011^4^. At the time of the accident, wheat and barley were in their young vegetative phases (having only a few fully expanded leaves)^5^. At 2 months after the accident, Nakanishi *et al*. measured the radioactivity level in leaves and found that it was highest in the senescent leaves that had been expanded at the time of the accident^5^. The radioactivity level gradually decreased towards the upper leaves. In addition, the lowest radioactivity level was found in the ears. Based on these results, it was hypothesized that the excess radioactivity (above the standard) in grains of wheat and barley was a result of the translocation of RCs deposited on the leaves to the grains as well as the absorption of the RCs deposited on the soil by the roots.

The decrease in RCs absorption by the roots and RCs translocation to the edible parts by increasing the soil exchangeable potassium (ExK) content was confirmed in some crops, including wheat^6^, as previously reported^7–11^. As a result of increasing potassium (K) applications to decrease RCs transfer from soil to grain, wheat samples that exceed the standard have not been detected since 2012. Increasing K applications has resulted in decreased RCs transfers from the soil to the plants of various species, such as rice (*Oryza sativa* L.)^12,13^, soybean (*Glycine max* L.)^14^, buckwheat (*Fagopyrum esculentum* Moench)^15–18^ and cypress (*Chamaecyparis obtusa* (Sieb. et Zucc.) Endl.)^19^ in the area affected by RCs.

With the objective of investigating the effects of genetic variation on RCs accumulation in crops, the rice core-collection has been analyzed in field experiments, revealing ~12-fold differences in RCs concentration in grains^20,21^. This allowed the identification of rice varieties having low RCs accumulation levels. Additionally, in soybean, Takagi *et al*. detected approximately 10-fold differences in stable cesium (^133^Cs) concentrations in grains of different varieties^22^. Low cesium-accumulating mutants have been acquired in rice^23,24^. In wheat, Schimmack *et al*. and Putyatin *et al*. reported varietal differences in RCs accumulation using 28 and 6 cultivars in Europe, respectively, after the Chernobyl accident^25,26^. However, compared with rice and soybean, only limited information is available on the effects of genetic diversity on RCs accumulation in wheat.

Determining the effects of varietal differences in RCs accumulation in wheat genetic resources will assist in the adoption of varieties with low RCs accumulation capacities in future breeding programs and in the construction of comprehensive countermeasures, to decrease RCs accumulation in wheat by combining K applications and low RCs-accumulating varieties. In this study, comparisons of Japanese landraces, commercial cultivars released in Japan and global genetic resources in terms of RCs accumulation, were conducted in a field located in Fukushima Prefecture for two growing seasons (2012–2013 and 2013–2014). We also investigated the effects of the variability in the soil RCs concentration and soil ExK content within a field and the differences in agronomic traits on the RCs accumulation in wheat varieties.

## Results

### Soil RCs concentration, soil ExK content and RCs accumulation in wheat

**Figure 1:**
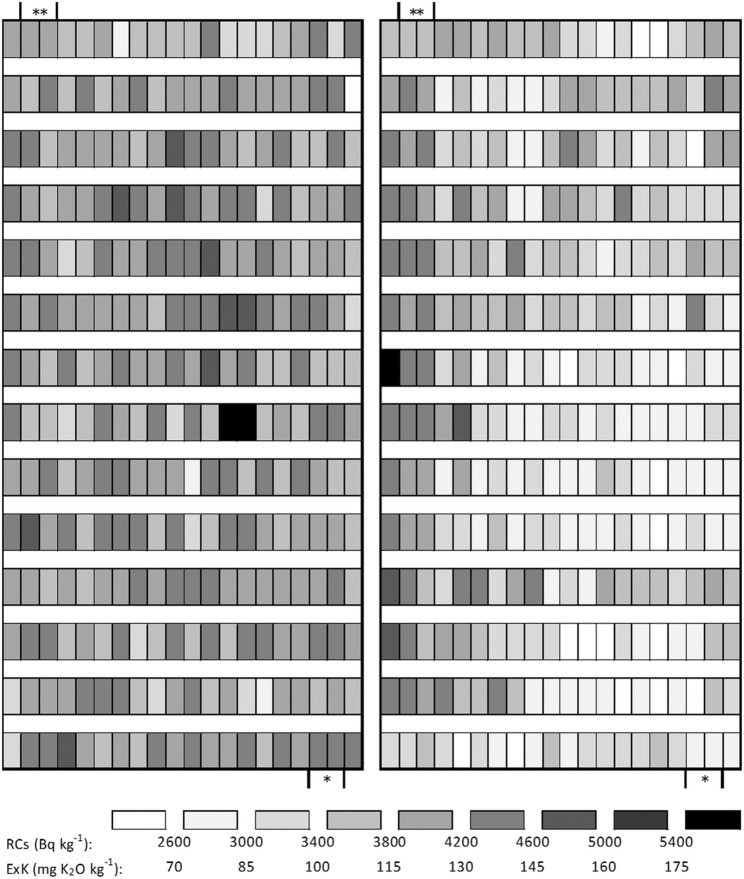
Distributions of RCs concentration (left) and ExK content (right) in the field at wheat harvest (2012–2013 growing season). Size of each plot is 2.0 m × 0.7 m. * and ** indicate the inlet and outlet for irrigation water, respectively, when the field was used as a rice paddy (before 2011).

Variations in the soil RCs concentrations and soil ExK contents are shown in Fig. 1. Soil RCs concentrations differed by approximately twofold in the field and ranged from 2,321 to 5,901 Bq kg^−1^ at the harvest in the 2012–2013 growing season. The average value and coefficient of variation (CV) were 4,085 Bq kg^−1^ and 0.12. The soil ExK contents differed by approximately fourfold and ranged from 51.6 to194.1 mg K_2_O kg^−1^ (from 42.8 to 161.1 mg K kg^−1^). The average value and CV were 100.2 mg K_2_O kg^−1^ (83.2 mg K kg^−1^) and 0.23.

The grain RCs concentrations of each variety in the 2012–2013 and 2013–2014 growing seasons ranged from 10.3 to 104.6 Bq kg^−1^ and from 8.8 to 71.4 Bq kg^−1^, respectively (Table 1). The shoot RCs concentration was higher than the grain RCs concentration, ranging from 24.6 to 285.8 Bq kg^−1^ in the 2012–2013 growing season and from 18.3 to 266.2 Bq kg^−1^ in the 2013–2014 growing season. The grain RCs to shoot RCs ratio (G-S RCs ratio), which indicates the RCs partitioning between shoot and grain, also showed a wide range of variation, ranging from 0.197 to 0.860 and from 0.187 to 0.760 in the 2012–2013 and 2013–2014 growing seasons, respectively. Transfer factor (TF), which is a major indicator of RCs translocation from the growing medium to the edible parts of plant^27^, was calculated by the following equation:

1 $${\rm{TF}}={\rm{grain}}\,{\rm{RCs}}\,{\rm{concentration}}\,({\rm{Bq}}\,{{\rm{kg}}}^{-1}{\rm{DW}})/{\rm{soil}}\,{\rm{RCs}}\,{\rm{concentration}}\,({\rm{Bq}}\,{{\rm{kg}}}^{-1}{\rm{DW}})$$

**Table 1:** Ranges, averages and coefficient of variations (CV) of RCs concentrations in plant parts, the G-S RCs ratios and TF^α^.

	Range	Average	CV
2012–2013 growing season (n = 234)			
Grain RCs concentration (Bq kg^−1^)	10.3–104.6	40.0	0.595
Shoot RCs concentration (Bq kg^−1^)	24.6–285.8	94.5	0.555
G-S RCs ratio	0.197–0.860	0.428	0.309
TF	0.002–0.027	0.010	0.603
2013–2014 growing season (n = 198)			
Grain RCs concentration (Bq kg^−1^)	8.8–71.4	37.2	0.343
Shoot RCs concentration (Bq kg^−1^)	18.3–266.2	92.9	0.369
G-S RCs ratio	0.187–0.760	0.420	0.291
TF	0.002–0.021	0.010	0.357

^α^RCs ratio of grain concentration (Bq kg^−1^) to soil concentration (Bq kg^−1^).

**Figure 2:**
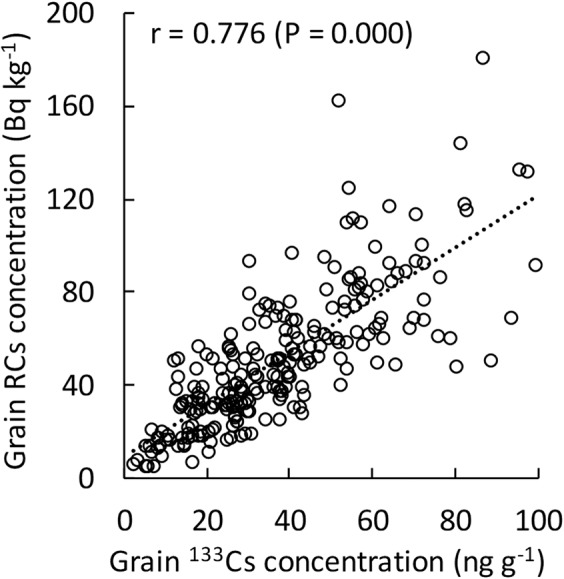
Relationship between stable (^133^Cs) concentration and RCs concentration in grain. n = 231 (subset in a replication of 2012–2013 growing season).

TF was ranged from 0.002 to 0.027 and from 0.002 to 0.021 in the 2012–2013 and 2013–2014 growing seasons, respectively. The average RCs concentrations in grain and shoot, as well as the G-S RCs ratio, were similar between the two growing seasons but slightly lower in the 2013–2014 growing season. Average TF stayed at the same level in both growing seasons. The CVs in grain and shoot RCs concentrations and TF were smaller in the 2013–2014 growing season compared with those in the 2012–2013 growing season. RCs concentration and ^133^Cs concentration in grain showed significant positive correlation in 2012–2013 growing season (Fig. 2).

**Figure 3:**
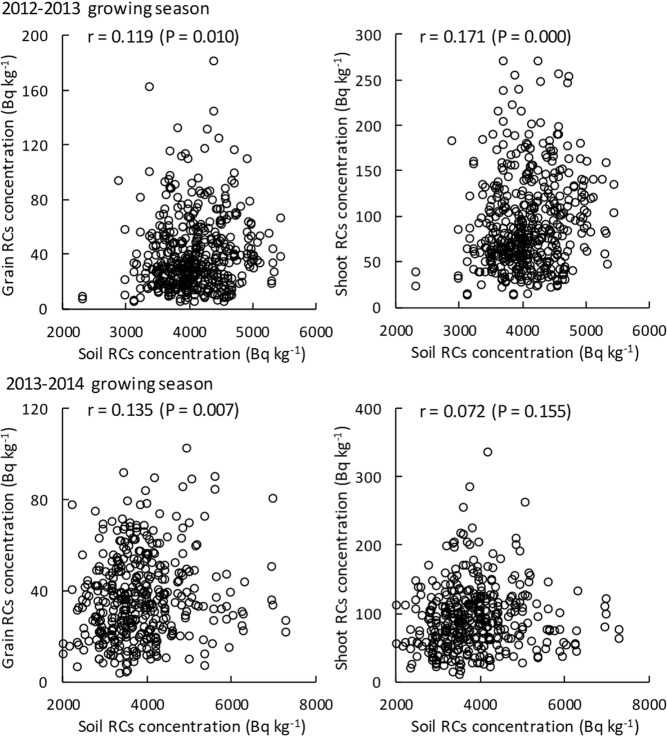
Correlations between soil RCs concentrations and both grain (left) and shoot (right) RCs concentrations. n = 468 (2012–2013 growing season) and 396 (2013–2014 growing season). Correlation coefficients were calculated using linear approximation.

In Fig. 3, correlations between the soil RCs concentration and the grain and shoot RCs concentrations are shown. In the 2012–2013 growing season, the correlations were significant despite the correlation coefficients being low. The plants grown in soil with a high RCs concentration tended to have high RCs concentrations in their grains and shoots. In the 2013–2014 growing season, the grain RCs concentration had a significant positive correlation with the soil RCs concentration. This was also true in the 2012–2013 growing season, but the relationship between the soil RCs concentration and shoot RCs concentration was not significant.

**Figure 4:**
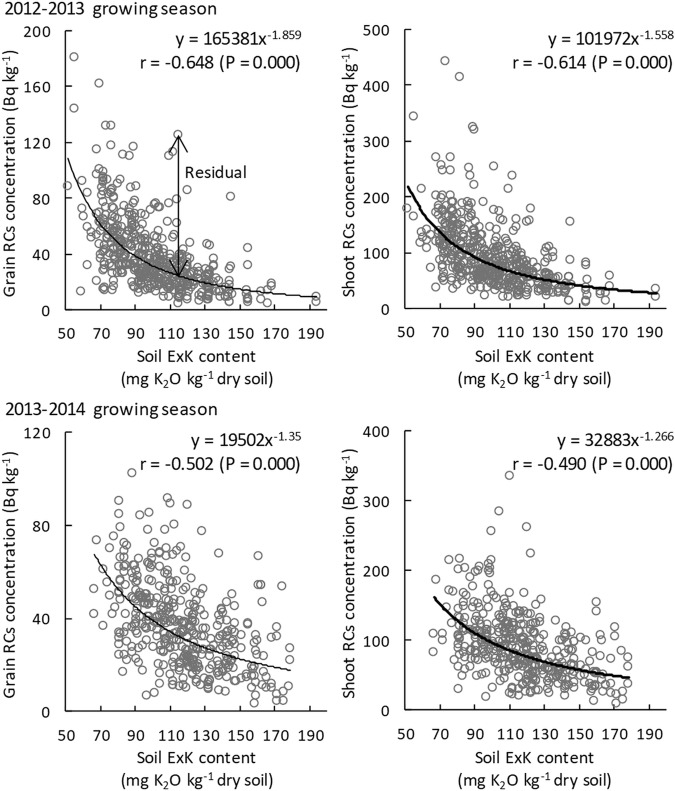
Correlations between soil ExK contents and both grain (left) and shoot (right) RCs concentrations. Residuals of the relationships between soil ExK content and grain/shoot RCs concentration were calculated for each sample. n = 468 (2012–2013 growing season) and 396 (2013–2014 growing season). Correlation coefficients were calculated using power approximation.

The soil ExK content was significantly negatively correlated with the grain and shoot RCs concentrations in both 2012–2013 and 2013–2014 growing seasons (Fig. 4), and the plants grown in soil with a high ExK content had low RCs concentrations in their grains and shoots. The correlation coefficients between the soil ExK content and RCs concentrations in the grain and shoot were relatively high compared with those between the soil RCs concentration and RCs concentrations in the grain and shoot.

### Analysis of genetic difference in RCs accumulation in wheat

**Figure 5:**
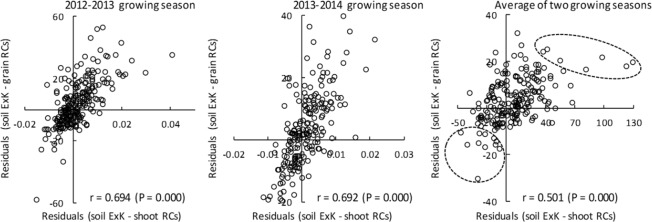
Correlations between shoot and grain residuals. n = 198. Correlation coefficients were calculated using linear approximation. Circled varieties were selected as having a high/low RCs accumulation.

To evaluate the RCs accumulation in each variety by reducing the effect of soil ExK, residuals of the relationships between the soil ExK content and RCs concentrations in grain residuals (soil ExK − grain RCs) and shoot residuals (soil ExK − shoot RCs) were calculated (Fig. 4), and the year-to-year correlations between the residuals were also analyzed. Although correlation coefficients between years for the RCs concentrations in grains (r = 0.082, P = 0.251, n = 198) and shoots (r = 0.109, P = 0.126, n = 198) were not significant, shoot residuals, indicators of how much the RCs accumulation in each variety reduced the effect of the soil ExK content, showed a significant positive year-to-year correlation with a small correlation coefficient value (r = 0.161, P = 0.023, n = 198). The grain to shoot RCs concentration ratio was significantly positively correlated between years (r = 0.587, P = 0.000, n = 198). Although the year-to-year correlation was not significant for the grain residuals (r = 0.040, P = 0.572, n = 198), there was a significant correlation between the grain and shoot residuals (Fig. 5).

The varieties with low or high RCs accumulation were selected according to the shoot and grain residuals (Fig. 5, Table 2). The grain RCs concentration in low RCs-accumulating varieties was less than half of those in high RCs-accumulating varieties. The soil-to-grain TF of RCs was also lower in varieties having low RCs accumulations (0.0062 on average) compared with those having high RCs accumulations (0.0147 on average). The G-S RCs ratio and the measured agronomic traits (days from sowing to heading (HD), culm length (CL), spike number per plant (SN), grain weight per plant (GW), 100 grain weight (100 GW) and grain number per spike (GN)) did not show clear differences between varieties having low and high RCs accumulation levels.

**Table 2:** Varieties with lowest/highest RCs accumulation levels as evaluated by residuals (soil ExK-shoot RCs and soil ExK-grain RCs)^α^.

Variety No.	Grain RCs concentration		Residual (soil ExK - grain RCs)		Residual (soil ExK - shoot RCs)		G-S RCs ratio		TF ^β^		HD^γ^	CL^δ^	SN^ε^	GW^ζ^	100 GW^η^	GN^θ^
	2012–2013	2013–2014	2012–2013	2013–2014	2012–2013	2013–2014	2012–2013	2013–2014	2012–2013	2013–2014						
*The 11 varieties with low residuals*																
10	14.1	15.3	−9.6	−12.0	−31.9	−24.0	0.47	0.34	0.0039	0.0037	204	84	4.11	6.29	3.11	48.7
40	13.8	18.7	−6.7	−19.4	−19.2	−6.6	0.42	0.21	0.0034	0.0045	204	81	5.57	8.62	3.03	50.3
52	15.2	29.2	−25.7	−2.6	−57.9	38.1	0.45	0.25	0.0039	0.0077	195	66	5.31	7.49	3.38	41.6
55	18.1	25.5	−17.7	−17.2	−6.4	−7.9	0.23	0.26	0.0037	0.0070	195	55	3.95	5.50	3.44	40.3
97	22.0	40.6	−25.7	−7.8	−36.8	−6.1	0.29	0.36	0.0060	0.0097	197	68	6.11	10.18	3.73	43.5
118	16.9	23.6	−5.7	−16.7	−17.5	−25.7	0.39	0.32	0.0048	0.0064	199	84	5.56	8.09	2.77	52.5
133	21.3	34.3	−12.1	−17.6	−30.2	−26.0	0.41	0.34	0.0061	0.0114	204	84	3.80	3.94	6.04	17.0
138	45.6	23.8	−12.4	−8.6	−48.5	−8.6	0.55	0.67	0.0108	0.0075	207	90	2.72	5.79	4.47	47.8
206	21.4	39.6	−33.5	2.2	−28.5	6.1	0.24	0.40	0.0058	0.0092	211	60	3.93	6.44	3.48	48.1
219	14.3	29.8	−57.7	−3.0	−68.7	9.5	0.20	0.32	0.0039	0.0084	200	56	1.14	1.48	3.85	31.3
221	19.2	15.4	−12.1	−19.1	−30.6	−49.7	0.45	0.42	0.0047	0.0037	199	69	4.46	9.50	3.65	59.6
*The 7 varieties with high residuals*																
6	55.6	48.0	23.5	24.7	29.8	45.1	0.51	0.46	0.0146	0.0159	197	73	6.24	10.66	3.45	49.0
28	55.1	46.7	21.8	13.9	157.9	87.4	0.24	0.28	0.0123	0.0094	197	52	5.83	8.41	3.08	46.3
66	53.3	40.9	22.7	10.3	105.6	57.1	0.29	0.31	0.0133	0.0119	203	59	4.83	8.46	3.34	52.8
76	52.1	69.1	6.7	32.3	84.1	174.6	0.28	0.26	0.0113	0.0179	198	56	5.00	7.54	3.66	41.2
99	104.6	49.2	38.3	11.8	61.9	20.9	0.47	0.43	0.0244	0.0142	206	88	4.41	6.24	3.85	36.7
111	45.7	50.6	12.3	28.2	37.8	72.8	0.37	0.39	0.0108	0.0144	196	51	4.76	5.67	2.84	42.2
207	85.4	54.8	35.2	8.3	171.1	25.7	0.30	0.39	0.0214	0.0136	213	77	1.22	1.49	4.18	29.2
	**^i^	**	**	**	**	**			**	**						

^α^The varieties with low/high grain RCs accumulation levels were selected from Fig. 5; ^β^RCs ratio of grain concentration (Bq kg^−1^) to soil concentration (Bq kg^−1^); ^γ^Days from sowing to heading; ^δ^Culm length; ^ε^Spike number per plant; ^ζ^Grain weight per plant; ^η^100 grain weight; ^θ^Grain number per spike; ^i^ **indicate significant difference between variety groups at P < 0.01.

### Relationships between RCs accumulation and agronomic traits

Table 3 shows the correlation coefficients between the RCs accumulation and the agronomic traits in all the varieties tested. The grain RCs concentration showed significant positive correlations with grain and shoot residuals (P = 0.000), shoot RCs concentration (P = 0.000), G-S RCs ratio (P = 0.000) and 100 GW (P < 0.05). Grain RCs concentration and shoot RCs concentration and grain residual had significant positive correlations with HD (P = 0.000). Grain RCs concentration, grain residual and the G-S RCs ratio had significant positive correlations with CL (P = 0.000). Grain residual was also positively correlated with SN (P = 0.005) and GW (P = 0.010). The G-S RCs ratio was positively correlated with 100 GW (P = 0.007) and negatively correlated with GN (P = 0.030).

**Table 3:** Correlation coefficients among RCs accumulation and agronomic traits (2012–2013 growing season).

	Residual (soil ExK - shoot RCs)	Residual (soil ExK - grain RCs)	Shoot RCs	Grain RCs	G-S RCs ratio	HD^α^	CL^β^	SN^γ^	GW^δ^	100GW^ε^	GN^ζ^
Residual (soil ExK - shoot RCs)		0.649**	0.853**	0.493**	−0.344**	0.126	−0.029	0.099	0.120	−0.062	0.091
Residual (soil ExK - grain RCs)	234^η^		0.555**	0.793**	0.391**	0.279**	0.347**	0.184**	0.168*	0.039	0.017
Shoot RCs	234	234		0.735**	−0.218**	0.244**	0.072	0.001	0.027	0.050	−0.002
Grain RCs	234	234	234		0.433**	0.401**	0.398**	0.045	0.034	0.166*	−0.103
G-S RCs ratio	234	234	234	234		0.260**	0.491**	0.066	0.020	0.175**	−0.142*
HD	234	234	234	234	234		0.641**	0.051	−0.007	0.338**	−0.278**
CL	234	234	234	234	234	234		0.200**	0.173**	0.324**	−0.147*
PN	234	234	234	234	234	234	234		0.844**	−0.370**	0.281**
GW	233	233	233	233	233	233	233	233		−0.29**	0.628**
100GW	233	233	233	233	233	233	233	233	233		−0.575**
GN	233	233	233	233	233	233	233	233	233	233	

^α^Days from sowing to heading; ^β^Culm length; ^γ^Spike number per plant; ^δ^Grain weight per plant; ^ε^100 grain weight; ^ζ^Grain number per spike; ^η^Analyzed number of varieties; ** and * indicate significance at P < 0.01 and P < 0.05, respectively.

## Discussion

The release of RCs due to the accident of nuclear accident brought to the growing crops both foliar uptake and root uptake of RCs. On the foliar uptake of RCs by the crops, precise experiments have been revealed that the ^134^Cs accumulation in edible parts is affected by the growth stage taken place RCs deposition in spring wheat and oilseed rape (*Brassica napus* L.)^28^. In the case of TEPCO’s FDNPP accident, it has been considered that the most of the radioactive materials emitted from TEPCO’s FDNPP are deposited or transported out of the Tohoku and Kanto regions (including Fukushima prefecture) within a few days and do not stay in the atmosphere in the regions^29^. RCs concentration in grain collected from 2012–2013 growing season showed significant positive correlation with ^133^Cs concentration in grain in this study (Fig. 2). ^133^Cs in the grain would be derived from root uptake. Accordingly, the majority of RCs accumulated in wheat in this study is considered to derive from root uptake.

Average values of soil-to-grain RCs TF in this study was 0.010 in both 2012–2013 and 2013–2014 growing seasons (Table 1). Typical TF for cereals are 0.021 (95% confidence intervals 0.0017–0.25), 0.014 (0.00045–0.42), 0.011 (0.00057–0.21) and 0.043 (0.0038–0.49) for sand, loam, clay and organic, respectively^30^. International Atomic Energy Agency has also reported the TF in cereals in temperate environment, which are 0.039 (minimum-maximum 0.002–0.66), 0.020 (0.0008–0.20), 0.011 (0.0002–0.09) and 0.043 (0.010–0.73) for sand, loam, clay and organic, respectively^31^. The field conducted our study was loamy clay texture, and the TF values were similar to those previously reported for clay and loam. As the factor affecting to RC transfer from soil to plant, soil exchangeable (plant-available) RCs concentration has had significant positive correlation with RCs concentration in buckwheat cultivated in the field in Fukushima prefecture^16^. Exchangeable RCs in soil can be adsorbed to the frayed edge sites of clay minerals^32–34^, and RCs adsorbed to clay minerals becomes difficult to be absorbed by roots of plant. Matsunaga *et al*. has surmised that the majority of ^137^Cs in the soil is fixed to the soil constituents within three months after the release of ^137^Cs into the environment by using the soils collected from croplands and grasslands in Fukushima prefecture^35^. In addition, Takeda *et al*. has also reported that the exchangeable ^137^Cs proportion in soils represents exponentially decrease (from 72% to 22%) during 100 days after the addition of soluble ^137^Cs, and is approximately constant after 100 days with the incubation experiment using the Andosol samples collected in Aomori prefecture in Japan^36^. Similar TFs in 2012–2013 and 2013–2014 growing seasons in this study would indicate that the rapid fixation of the majority of RCs had completed in the field at the start of experiment (November, 2012). On the other hand, Manaka *et al*. has observed the moderate decreasing trends in proportion of exchangeable ^137^Cs over six years in Fukushima forest soils^37^. Consequently, it is expected that the TF in the field used in this study is kept at constant level with moderate decrease in case of wheat cultivation with similar conditions in the future. It has also been revealed that soil-to-grain RCs transfer is affected by the other soil properties such as ExK content, organic matter, CEC, pH etc^38,39^.

The soil RCs concentration and ExK content in the field measured every 1.4 m^2^ (bulk soil collected at 10 points per area, with 280 total areas) showed approximately twofold (CV = 0.12) and fourfold (CV = 0.23) differences, respectively (Figs. 1, 3 and 4). Saito *et al*. reported that the RCs deposition in soil was heterogeneous, varying greatly even within a small area (9.0 m^2^, n = 5), and the CVs of the RCs concentrations for five soil samples collected at one location averaged 0.36^40^. Tsuiki and Maeda also showed large variations in soil RCs concentrations in a particular pasture (CV = 0.12, 144 m^2^, n = 144) and meadow (CV = 0.08, 500 m^2^, n = 25)^41^. The field used in this study could have had considerable variation in RCs concentration. However, the variation might have been reduced by tillage prior to cultivation^18^. Blanchet *et al*. suggested that the distribution of available soil K forms in the upper soil layer (0–20 cm) is influenced by land uses, such as agricultural practices^42^. Before the accident at the TEPCO’s FDNNP in 2011, the experimental field used in this study was a rice paddy field. In paddy fields, irrigation water flows from the inlet to outlet. The soil ExK content was relatively low around the irrigation water inlet. The variability in the soil ExK content may reflect the past flow of the irrigation water in this field.

The RCs concentrations in grain and shoot showed approximately 10-fold differences among varieties in this study (Table 1). The range of the differences was similar to those observed in rice^20,21^ and soybean (evaluation of ^133^Cs)^22^. However, RCs concentrations in grain and shoot had significant correlations with the soil RCs concentration (Fig. 3) and soil ExK content (Fig. 4). Centofanti and Frossard reported that a heterogeneous distribution of soil RCs affects the uptake and translocation of RCs by maize (*Zea mays* L.) roots^43^. Saito *et al*., Smolders *et al*. and Sotome *et al*. showed that increases in the ExK in soil result in a decrease in the RCs accumulation of a single wheat variety^6,44,45^. In this study, we showed that the correlation coefficients between soil ExK and RCs concentrations in grain and shoot were relatively high, which indicates that the soil ExK content has a more significant effect on RCs accumulation compared with wheat varietal differences.

By analyzing the grain and shoot residuals, we demonstrated that the shoot residuals were significantly correlated between years. The result indicates that the varietal differences in RCs accumulation are discernible only when considering the effects of the soil ExK content. Although the year-to-year correlation of grain residuals was not significant, the correlations between shoot and grain residuals were highly significant (Fig. 5). From these analyses, varieties with low or high RCs accumulation levels in grain were distinguished (Table 2). In general, varieties with low residuals had low grain RCs concentrations. Varieties No. 10 and 118, having low RCs accumulation levels, had the same variety name ‘Fukoku’, indicating that these are the same variety in different gene banks. The grain RCs concentration in ‘Fukoku’ was approximately half of that in varieties No. 85 (Nambu Komugi) and 86 (Kitakami Komugi), which are major varieties grown in eastern Japan, including Fukushima as shown in supplementary dataset (see Supplementary Table S2). Varieties with low RCs accumulation levels, such as ‘Fukoku’, will be useful for future analyses of the mechanisms regulating low RCs accumulation in wheat.

The grain RCs concentration showed a significant positive correlation with the shoot RCs concentration (Table 3). However, there were approximate fourfold differences in the G-S RCs ratios among varieties in both years, and a positive correlation between years. These results indicate that there are varietal differences in transportability of RCs from shoot to grain. Similar results were found for cadmium translocation in wheat^46^. The varieties with low and high residuals in Table 2 both had wide ranges of G-S RCs ratios. For example, variety No. 28 had a high RCs concentration in the grain but had a relatively low G-S RCs ratio. Such varieties may have high RCs absorption capabilities and low transportability of RCs from shoot to grain. Thus, RCs absorption and translocation appear to be controlled by different mechanisms in wheat, and RCs accumulation in the grain should be analyzed by carefully discriminating between absorption and translocation. The various mechanisms of RCs uptake in relation to K transporters have been reviewed^1,8^, and recently, several possible transporters involved in regulating RCs uptake have been reported^23,24,47^. Kojima *et al*. also detected large variations among rice varieties in RCs translocation from shoot to grain^20^. Feller *et al*. suggested that the transport of Cs from the xylem to phloem in wheat shoots is important for the accumulation of Cs in maturing wheat grains^48^. Different alleles of transporters among wheat varieties probably affect not only RCs absorption from the soil, but also translocation within plants, resulting in different final amounts of RCs accumulation in different varieties.

Some agronomic traits showed variation (see Supplementary Table S3), and had significant correlations with traits related to RCs accumulation (Table 3), although the former did not show clear differences between varieties with low and high residuals (Table 2). HD had a significant positive correlation with grain residual, grain RCs concentration and shoot RCs concentration (P < 0.01) (Table 3). Takagi *et al*. also reported a significant correlation between the grain^133^Cs concentration and days to flowering in a mini-core collection of soybean^22^. A longer HD may result in longer plant lives, and therefore, correlated with greater RCs absorption levels. The wheat varieties with longer HDs tended to have higher CL and larger 100 GW values in this study (Table 3). Significant positive correlations of CL with grain residual, grain RCs and the G-S RCs ratio indicate that a higher CL was correlated with a greater source of RCs for translocation to the grain. In addition, even though correlation coefficients were low, varieties with greater SNs had greater GN and lower 100 GW values, which were correlated with lower grain RCs concentrations and lower G-S RCs ratios (Table 3). These relationships may suggest that the varieties with greater GNs tended to contain smaller amounts of RCs per grain than those with smaller GNs. These results also suggest that the RCs concentration in the grain can be reduced not only by genetic improvement, but also by agricultural management. For example, by increasing the SN as discussed related to cadmium accumulation in wheat^49^.

In conclusion, wheat varieties with distinctive RCs concentrations in their grains were identified, although the RCs concentration in grain was affected by the variability in the soil RCs concentration and soil ExK content. The comparison of the grain RCs concentration and G-S RCs ratio among distinctive varieties indicated the independent control of RCs absorption and translocation. Thus, there appears to be varietal differences in RCs absorption and translocation in wheat germplasm. This indicates that genetic improvement to decrease grain RCs concentrations should be possible. Varieties with distinctive grain RCs accumulation capacities that were identified in this study will be useful for growing in RCs-affected areas, for analyses of mechanisms regulating RCs accumulation in wheat, and for breeding varieties with low RCs accumulation capacities. Adding the cultivation of low RCs-accumulating varieties to the increased application of K fertilizers could lead to decreasing RCs accumulation in crops.

Challenges in resuming agriculture in areas in which evacuation orders have been lifted are in progress^18^. Increasing the understanding of RCs translocation from soil to crops is ongoing and important for the resumption of agricultural practices and the reconstruction of communities in these areas.

## Materials and Methods

### Cultivation, agronomic trait evaluation and sampling of wheat in the field

In total, 234 wheat (*Triticum* spp.) varieties were used in this study (see Supplementary Table S1). Varieties No. 1–95 represented the core collection of Japanese wheat varieties^50^. Varieties No. 96–234 were from the National BioResource Project of Japan (http://shigen.nig.ac.jp/wheat/komugi/)^51,52^. These varieties were grown in 2012–2013 growing season in a rotational upland field (about 800 m^2^) in Fukushima Prefecture that had been exposed to RCs deposition in 2011. In 2013–2014 growing season, 198 of these varieties were again investigated in the same field. The sowing dates were 2 November and 31 October in 2012 and 2013, respectively. The plots were 1.0-m long single rows in the field for both seasons. The planting distance was 70 cm between rows and 5 cm between plants. The experimental design was a randomized block with two replications per variety in both years. Fertilizer was applied just before seeding: 35 and 35 kg ha^−1^ of N and P_2_O_5_, respectively, after the application of 1 t ha^−1^ of magnesium lime. K_2_O was not applied in this study because Cs uptake in wheat is decreased by increasing K concentrations in media^7,9–11^. In the 2012–2013 growing season, the day of heading for each variety was determined, and the HD were calculated. The CL and SN were also measured from heading to maturity. When each variety reached maturity, all the plants of each plot (1.0 m × 0.7 m) were harvested by cutting the culm at 5 cm above the ground level. After harvesting, soil samples at 20-cm depths from every other plot (2.0 m × 0.7 m) were collected from around the plant roots with a round worm scoop (Fujiwara Scientific Company Co., Ltd., Tokyo, Japan). Soil collections using the scoop were conducted 10 times per sampling (1.4 m^2^), and then mixed to form one sample.

### Sample preparation and analyses

For the grain samples, total grain weight was recorded after threshing and winnowing. Additionally, 100GWs were measured three times for each sample, and the GN were calculated. Grain RCs (^134^Cs and ^137^Cs) concentrations were measured using a germanium semiconductor detector (GC2520–7500SL; Canberra Japan KK, Tokyo, Japan) as described in Kubo *et al*.^16^. The RCs concentration measurement method had a 10% error range. Grain ^133^Cs concentration of subset of materials (grain of varieties in a replication of 2012–2013 growing season) were determined using an inductively coupled plasma mass spectrometer (ICP-MS, 7700x; Agilent Technologies, Santa Clara, CA, USA). Sample preparation for ICP-MS analysis was conducted as described in Kubo *et al*.^46^. After washing the wheat plants with tap water to avoid soil contamination^53^, the samples were cut into small pieces, and dried for 48 h at 80 °C in a ventilated oven. Dried samples were ground into a powder using a blender (D3V-10, Osaka Chemical Co., Osaka, Japan) for the measurement of RCs concentration with a germanium semiconductor detector as described above. Soil samples were dried at 40 °C for 7 d. They were ground to less than 2.0-mm particles using a Dust Shield Automatic Mill and Screen for Soil RK4II (DIK-2610; Daiki Rika Kogyo Co. Ltd., Saitama, Japan) for the measurement of RCs concentration and ExK content. Soil RCs concentration were determined using a germanium semiconductor detector as described for the plant samples. Soil-to-grain RCs TF was calculated by Eq. (1). Soil ExK was extracted from soil samples in 1 M ammonium acetate (HN_4_OAc) at a 1:10 soil:solution ratio by shaking for 1 h. The soil ExK content was determined using atomic absorption spectrophotometry (ZA3000, Hitachi High-Tech Science, Tokyo, Japan).

### Statistical analyses

Pearson’s correlation analyses and Student’s t-test were conducted using SPSS software (IBM SPSS ver. 25 for Windows; International Business Machine Co., USA). Residuals of the relationships between traits were calculated using software (Excel application in Microsoft Office 365 ProPlus; Microsoft Co., USA).

## Supplementary information

Supplementary materials.

## Data Availability

The data sets generated or analyzed during the current study are available from the supplementary information. The materials used in this study are available from the National BioResource Project/WHEAT (https://shigen.nig.ac.jp/wheat/komugi/strains/aboutNbrp.jsp) and Genebank Project, NARO (https://www.gene.affrc.go.jp/index_en.php).

## References

[1] Burger A, Lichtscheidl I (2018). Stable and radioactive cesium: A review about distribution in the environment, uptake and translocation in plants, plant reactions and plants’ potential for bioremediation. Sci. Total. Environ..

[2] Ohara T, Morino Y, Tanaka A (2011). Atmospheric behavior of radioactive materials from Fukushima Daiichi Nuclear Power Plant. J. Natl. Inst. Public. Health.

[3] MHLW. Radioactive Materials in Foods – Current Situation and Protective Measures http://www.mhlw.go.jp/english/topics/2011eq/dl/food-130926_1.pdf (2012).

[4] MAFF. Results of inspections on radioactivity levels in agricultural product http://www.maff.go.jp/e/policies/food_safety/h30gaiyo.html (2019)

[5] Nakanishi TM, Kobayashi NI, Tanoi K (2013). Radioactive cesium deposition on rice, wheat, peach tree and soil after nuclear accident in Fukushima. J. Radioanal. Nucl. Chem..

[6] Saito, T. *et al*. Suppressive countermeasures of radiocesium uptake in crop by potassium fertilizer application. *Abstract of the 240*^*th*^*Meeting of the Crop Science Society of Japan*, 280–281; 10.14829/jcsproc.241.0_280 (In Japanese with English title) (2016).

[7] Zhu YG, Shaw G, Nisbet AF, Wilkins BT (1999). Effects of external potassium supply on compartmentation and flux characteristics of radiocaesium in intact spring wheat roots. Ann. Bot..

[8] White PJ, Broadley MR (2000). Mechanisms of caesium uptake by plants. N. Phytol..

[9] Zhu YG, Shaw G, Nisbet AF, Wilkins BT (2000). Effect of potassium starvation on the uptake of radiocaesium by spring wheat (*Triticum aestivum* cv. Tonic). Plant. Soil..

[10] Zhu YG, Smolders E (2000). Plant uptake of radiocaesium: a review of mechanisms, regulation and application. J. Exp. Bot..

[11] Zhu YG (2001). Effect of external potassium (K) supply on the uptake of ^137^Cs by spring wheat (*Triticum aestivum* cv. Tonic): a large-scale hydroponic study. J. Environ. Radioact..

[12] Kato N (2015). Potassium fertilizer and other materials as countermeasures to reduce radiocesium levels in rice: Results of urgent experiments in 2011 responding to the Fukushima Daiichi Nuclear Power Plant accident. Soil. Sci. Plant. Nutr..

[13] Ishikawa J (2018). Dynamic changes in the Cs distribution throughout rice plants during the ripening period, and effects of the soil-K level. Plant. Soil..

[14] Hirayama T, Takeuchi M, Nakayama H, Nihei N (2018). Effects of decreasing radiocesium transfer from the soil to soybean plants and changing the seed nutrient composition by the increased application of potassium fertilizer. Bull. Fukushima Agric. Tech. Cent..

[15] Kubo K (2015). Analyses and countermeasures for decreasing radioactive cesium in buckwheat in areas affected by the nuclear accident in 2011. Field Crop. Res..

[16] Kubo K, Fujimura S, Kobayashi H, Ota T, Shinano T (2017). Effect of soil exchangeable potassium content on cesium absorption and partitioning in buckwheat grown in a radioactive cesium-contaminated field. Plant. Prod. Sci..

[17] Kubo K (2018). Potassium behavior and clay mineral composition in the soil with low effectiveness of potassium application. Soil. Sci. Plant. Nutr..

[18] Kubo K, Kobayashi H, Fujimoto R, Ota T, Shinano T (2019). Towards the partial resumption of agriculture with buckwheat cultivation in fields physically decontaminated of radioactive cesium after the nuclear power plant accident in 2011: a case study in Yamakiya District, Fukushima. Plant. Prod. Sci..

[19] Komatsu M, Hirai K, Nagakura J, Noguchi K (2017). Potassium fertilisation reduces radiocesium uptake by Japanese cypress seedlings grown in a stand contaminated by the Fukushima Daiichi nuclear accident. Sci. Rep..

[20] Kojima K (2017). Characterization of 140 Japanese and world rice collections cultivated in Nihonmatsu-city in Fukushima in terms of radiocesium activity concentrations in seed grains and straws to explore rice cultivars with low radiocesium accumulation. J. Radioanal. Nucl. Chem..

[21] Ohmori Y (2014). Difference in cesium accumulation among rice cultivars grown in the paddy field in Fukushima Prefecture in 2011 and 2012. J. Plant. Res..

[22] Takagi K, Kaga A, Ishimoto M, Hajika M, Matsunaga T (2015). Diversity of seed cesium accumulation in soybean mini-core collections. Breed. Sci..

[23] Ishikawa S (2017). Low-cesium rice: mutation in *OsSOS2* reduces radiocesium in rice grains. Sci. Rep..

[24] Rai H (2017). Cesium uptake by rice roots largely depends upon a single gene, *HAK1*, which encodes a potassium transporter. Plant. Cell Physiol..

[25] Schimmack W (2004). Soil-to-grain transfer of fallout ^137^Cs for 28 winter wheat cultivars as observed in field experiments. Radiat. Environ. Biophys..

[26] Putyatin YV, Seraya TM, Petrykevich OM, Howard BJ (2006). Comparison of the accumulation of ^137^Cs and ^90^Sr by six spring wheat varieties. Radiat. Environ. Biophys..

[27] IAEA (1982). Generic models and parameters for assessing the environmental transfer of radionuclides from routine releases. Saf. Ser..

[28] Bengtsson SB, Eriksson J, Gärdenäs AI, Vinichuk M, Rosén K (2013). Accumulation of wet-deposited radiocaesium and radiostrontium by spring oilseed rape (*Brássica napus* L.) and spring wheat (*Tríticum aestívum* L.). Environ. Poll..

[29] Morino Y, Ohara T, Nishizawa M (2011). Atmospheric behavior, deposition, and budget of radioactive materials from the Fukushima Daiichi nuclear power plant in March 2011. Geophys. Res. Lett..

[30] Nisbet AF, Woodman RFM (2000). Soil-to-plant transfer factors for radiocesium and radiostrontium in agricultural systems. Health Phys..

[31] IAEA (2010). Handbook of parameter values for the prediction of radionuclide transfer in terrestrial and freshwater environments. Technical Rep. Ser..

[32] Sawhney BL (1972). Selective sorption and fixation of cations by clay minerals: A review. Clays Clay Miner..

[33] Mukai H (2016). Cesium adsorption/desorption behavior of clay minerals considering actual contamination conditions in Fukushima. Sci. Rep..

[34] Okumura M (2019). Radiocesium interaction with clay minerals: Theory and simulation advances Post–Fukushima. J. Environ. Radioact..

[35] Matsunaga T (2013). Comparison of the vertical distributions of Fukushima nuclear accident radiocesium in soil before and after the first rainy season, with physicochemical and mineralogical interpretations. Sci. Total. Environ..

[36] Takeda A, Tsukada H, Nakao A, Takaku Y, Hisamatsu S (2013). Time-dependent changes of phytoavailability of Cs added to allophanic Andosols in laboratory cultivations and extraction tests. J. Environ. Radioact..

[37] Manaka T (2019). Six-year trends in exchangeable radiocesium in Fukushima forest soils. J. Environ. Radioact..

[38] Djungova R, Kovacheva P, Todorov B, Zlateva B, Kuleff I (2005). On the influence of soil properties on the transfer of ^137^Cs from two soils (Chromic Luvisol and Eutric Fluvisol) to wheat and cabbage. J. Environ. Radioact..

[39] Monira B, Ullah SM, Mollah AS, Chowdhury N (2005). ^137^Cs-uptake into wheat (*Triticum Vulgare*) plants from five representative soils of Bangladesh. Environ. Monit. Assess..

[40] Saito K (2015). Detailed deposition density maps constructed by large-scale soil sampling for gamma-ray emitting radioactive nuclides from the Fukushima Dai-ichi Nuclear Power Plant accident. J. Environ. Radioact..

[41] Tsuiki M, Maeda T (2012). Spatial variability of radioactive cesium fallout on grasslands estimated in various scales. Grassl. Sci..

[42] Blanchet G (2017). Spatial variability of potassium in agricultural soils of the canton of Fribourg, Switzerland. Geoderma.

[43] Centofanti T, Frossard E (2006). Uptake and translocation of ^134^Cs by maize roots as affected by heterogeneous distribution of ^134^Cs. Plant. Soil..

[44] Smolders E, Kiebooms L, Buysse J, Merckx R (1996). ^137^Cs uptake in spring wheat (*Triticum aestivum* L. cv. Tonic) at varying K supply. Plant. Soil..

[45] Sotome, T. *et al*. Transfer of radioactive cesium in wheat and barley at Tochigi prefecture. *Abstract of the 237*^*th*^*Meeting of the Crop Science Society of Japan*, 402; 10.14829/jcsproc.237.0_402 (In Japanese with English title) (2014).

[46] Kubo K (2016). Varietal differences in the absorption and partitioning of cadmium in common wheat (*Triticum aestivum* L.). Environ. Exp. Bot..

[47] Nieves-Cordones M (2017). Production of low-Cs^+^ rice plants by inactivation of the K+ transporter OsHAK1 with the CRISPR-Cas system. Plant. J..

[48] Feller U, Riesen T, Zehnger HJ (2000). Transfer of cesium from the xylem to the phloem in the stem of wheat. Biol. Plant..

[49] Kubo K (2008). Cadmium concentration in grains of Japanse wheat cultivars: genotypic difference and relationship with agronomic characteristics. Plant. Prod. Sci..

[50] Kojima H (2017). Development and evaluation of the core collection of Japanese wheat varieties. Bull. NARO Crop. Sci..

[51] Takenaka S, Nitta M, Kawahara T, Nasuda S (2014). “Core-collection” project of the National BioResource Project-Wheat, Japan: 2013 progress report. Wheat Inf. Serv..

[52] Takenaka S, Nitta M, Nasuda S (2018). Population structure and association analyses of the core collection of hexaploid accessions conserved *ex situ* in the Japanese gene bank NBRP-Wheat. Gene Genet. Syst..

[53] Kubo K (2016). Decreasing radioactive cesium in lodged buckwheat grain after harvest. Plant. Prod. Sci..

