# Supplementary material for: Variations in radioactive cesium accumulation in wheat germplasm from fields affected by the 2011 Fukushima nuclear power plant accident
Source: Sci Rep. 2020 Feb 28;10:3744. doi: 10.1038/s41598-020-60716-w (PMC7048790; doi:10.1038/s41598-020-60716-w)
Supplement: Supplementary file 1 — Supplementary materials. [file 41598_2020_60716_MOESM1_ESM.pdf]

Supplementary Material for

Variations in radioactive cesium accumulation in wheat germplasm from fields affected by the 2011 Fukushima nuclear power plant accident

Katashi Kubo[1*], Hiroyuki Kobayashi[1,2], Miyuki Nitta[3], Shotaro Takenaka[3,4], Shuhei Nasuda[3], Shigeto Fujimura[1], Kyoko Takagi[1], Osamu Nagata[1], Takeshi Ota[1,5], Takuro Shinano[1,6]

[1] Agricultural Radiation Research Center, Tohoku Agricultural Research Center, National Agriculture and Food Research Organization (NARO), 50 Harajukuminami, Arai, Fukushima 960-2156, Japan
[2] Biodiversity Division, National Institute for Agro-Environmental Sciences, NARO, 3-1-3 Kannondai, Tsukuba, Ibaraki 305-8604, Japan
[3] Laboratory of Plant Genetics, Graduate School of Agriculture, Kyoto University, Kitashirakawaoiwake-cho, Sakyo-ku, Kyoto 606-8502, Japan
[4] Faculty of Agriculture, Ryukoku University, 1-5 Yokotani,Seta Oe-cho,Otsu,Shiga 520-2194, Japan
[5] Bio-oriented Technology Research Advancement Institution, NARO, 8 Higashida-cho, Kawasaki, Kanagawa 210-0005, Japan
[6] Laboratory of Plant Nutrition, Research Faculty of Agriculture, Hokkaido University, Kita 9 Nishi 9, Kita-ku, Sapporo, Hokkaido 060-8589, Japan

*Correspondence: K. Kubo, E-mail, ktskubo@affrc.go.jp

This PDF file includes
Supplementary Table S1
Supplementary Table S2
Supplementary Table S3

Supplementary Table S1. Varieties used in the study.

Variety No.	ID	Variety (Cultivar) name / Species	Country	Locarity
1	JWC 01	Akagawa Aka$^{\alpha}$	Japan	Hokkaido
2	JWC 02	Shiro Hada$^{\alpha}$	Japan	Hokkaido
3	JWC 03	Douson 1$^{\alpha}$	Japan	Hokkaido
4	JWC 04	Sapporo Haru Komugi$^{\alpha}$	Japan	Hokkaido
5	JWC 05	Soushuu 2$^{\alpha}$	Japan	Aomori
6	JWC 06	Shisen 1$^{\alpha}$	Japan	Iwate
7	JWC 07	Sairai Flutz	Japan	Iwate
8	JWC 08	Nishimura$^{\alpha}$	Japan	Yamagata
9	JWC 09	Shirasaya	Japan	Ibaraki
10	JWC 10	Fukoku$^{\alpha}$	Japan	Ibaraki
11	JWC 11	Akagara Ibaraki 1$^{\alpha}$	Japan	Ibaraki
12	JWC 12	Shiro Sanjaku$^{\alpha}$	Japan	Ibaraki
13	JWC 13	Aka Boro 1	Japan	Tochigi
14	JWC 14	Nitta Wase$^{\alpha}$	Japan	Gunma
15	JWC 15	Sunekiri 15$^{\alpha}$	Japan	Gunma
16	JWC 16	Aka Bouzu$^{\alpha}$	Japan	Saitama
17	JWC 17	Hosogara	Japan	Chiba
18	JWC 18	Shiro Daruma	Japan	Kanagawa
19	JWC 19	Aka Daruma$^{\alpha}$	Japan	Kanagawa
20	JWC 20	Wase Komugi$^{\alpha}$	Japan	Kanagawa
21	JWC 21	Aka Komugi	Japan	Yamanashi
22	JWC 22	Shibu Shirazu$^{\alpha}$	Japan	Nagano
23	JWC 23	Koshigun Zairaishu$^{\alpha}$	Japan	Niigata
24	JWC 24	Shiro Chabo$^{\alpha}$	Japan	Mie

Supplementary Table S1. (Continued).

Variety No.	ID	Variety (Cultivar) name / Species	Country	Locarity
25	JWC 25	Sakobore[α]	Japan	Shizuoka
26	JWC 26	Shin Chuunaga[α]	Japan	Hyogo
27	JWC 27	Hatakeda Komugi	Japan	Okayama
28	JWC 28	Yuusyouki 347[α]	Japan	Okayama
29	JWC 29	Hiroshima Shipuree[α]	Japan	Hiroshima
30	JWC 30	Mubou Chinko[α]	Japan	Hiroshima
31	JWC 31	Hiraki Komugi[α]	Japan	Tottori
32	JWC 32	Naka Soushuu[α]	Japan	Kagawa
33	JWC 33	Houman[α]	Japan	Tochigi
34	JWC 34	Sekichiku 1[α]	Japan	Aichi
35	JWC 35	Eshima Shinriki[α]	Japan	Fukuoka
36	JWC 36	Iga Chikugo[α]	Japan	Saga
37	JWC 37	Shiro Komugi[α]	Japan	Saga
38	JWC 38	Chikuzen[a]	Japan	Saga
39	JWC 39	Ichigou Haya Komugi[α]	Japan	Kumamoto
40	JWC 40	Shiro Bunbu[α]	Japan	Kumamoto
41	JWC 42	Sotome[α]	Japan	Nagasaki
42	JWC 43	Sada Bouzu[α]	Japan	Miyazaki
43	JWC 44	Nobeokabouzu Komugi[α]	Japan	Miyazaki
44	JWC 45	Sakigake 1[α]	Japan	Kagoshima
45	JWC 46	Akasabi Shirazu 1[α]	Japan	Hokkaido
46	JWC 47	Honkei 275[α]	Japan	Hokkaido
47	JWC 48	Hokkai 240[α]	Japan	Hokkaido
48	JWC 49	Kounosu 25[α]	Japan	Saitama

Supplementary Table S1. (Continued).

Variety No.	ID	Variety (Cultivar) name / Species	Country	Locarity
49	JWC 50	Saitama 27[α]	Japan	Saitama
50	JWC 51	Kantou 107	Japan	Ibaraki
51	JWC 52	Iga Chikugo Oregon[α]	Japan	Nagano
52	JWC 53	Gokuwase 4-15[α]	Japan	Hiroshima
53	JWC 54	Komugi Nourin 1[α]	Japan	Iwate
54	JWC 55	Harumaki Komugi Nourin 3[α]	Japan	Hokkaido
55	JWC 56	Komugi Nourin 9[α]	Japan	Aichi
56	JWC 57	Komugi Nourin 10[α]	Japan	Iwate
57	JWC 58	Komugi Nourin 16	Japan	Gunma
58	JWC 59	Komugi Nourin 26[α]	Japan	Nara
59	JWC 60	Komugi Nourin 27[α]	Japan	Iwate
60	JWC 61	Komugi Nourin 39	Japan	Iwate
61	JWC 62	Koushitsu Komugi Nourin 42[α]	Japan	Gunma
62	JWC 63	Komugi Nourin 50[α]	Japan	Gunma
63	JWC 64	Komugi Nourin 53[α]	Japan	Aichi
64	JWC 65	Komugi Nourin 55[α]	Japan	Iwate
65	JWC 66	Komugi Nourin 61[α]	Japan	Saga
66	JWC 67	Komugi Nourin 67[α]	Japan	Gunma
67	JWC 68	Harumaki Komugi Nourin 75	Japan	Hokkaido
68	JWC 69	Hokuei[α]	Japan	Hokkaido
69	JWC 70	Muka Komugi[α]	Japan	Hokkaido
70	JWC 71	Horoshiri Komugi[α]	Japan	Hokkaido
71	JWC 72	Takune Komugi[α]	Japan	Hokkaido
72	JWC 73	Haruhikari[α]	Japan	Hokkaido

Supplementary Table S1. (Continued).

Variety No.	ID	Variety (Cultivar) name / Species	Country	Locarity
73	JWC 74	Aoba Komugi[α]	Japan	Iwate
74	JWC 75	Hitsumi Komugi[α]	Japan	Iwate
75	JWC 76	Furutsu Masari[α]	Japan	Iwate
76	JWC 77	Yukichabo[α]	Japan	Niigata
77	JWC 78	Hikari Komugi[α]	Japan	Niigata
78	JWC 79	Fukuho Komugi[α]	Japan	Saitama
79	JWC 80	Fukuwase Komugi[α]	Japan	Hiroshima
80	JWC 81	Abukuma Wase	Japan	Fukuoka
81	JWC 82	Hokushin[α]	Japan	Hokkaido
82	JWC 83	Chihoku Komugi[α]	Japan	Hokkaido
83	JWC 84	Haruyutaka[α]	Japan	Hokkaido
84	JWC 85	Nanbu Komugi[α]	Japan	Iwate
85	JWC 86	Kitakami Komugi[α]	Japan	Iwate
86	JWC 87	Shirane Komugi[α]	Japan	Nagano
87	JWC 88	Bandou Wase[α]	Japan	Ibaraki
88	JWC 89	Shirogane Komugi[α]	Japan	Fukuoka
89	JWC 90	Chikugoizumi[α]	Japan	Fukuoka
90	JWC 91	Komugi Nourin 20[α]	Japan	Saga
91	JWC 92	Fujimi Komugi[α]	Japan	Saitama
92	JWC 93	Shirasagi Komugi[α]	Japan	Hiroshima
93	JWC 94	Junrei Komugi	Japan	Kagawa
94	JWC 95	Hachiman Komugi[α]	Japan	Iwate
95	JWC 96	Chinese Spring[α]	China	
96	KU-166	albidum Alef.[α]	China	Inner Mongoria Expedition,

Supplementary Table S1. (Continued).

Variety No.	ID	Variety (Cultivar) name / Species	Country	Locarity
97	KU-265	hostianum-compactoides Goekg. (Norin 10)$^{\alpha}$	Japan	Collection of Lab. of Plant Breeding, Facul. of Agr., Kyoto Univ.,
98	KU-309	graecum Koern. (Baart)$^{\alpha}$	USA	Collection of Univ. Wash., Pullman,
99	KU-333	- (Selkerk)$^{\alpha}$	Canada	Collection of Univ. Manitoba,
100	KU-336	erythrospermum Koern. (Wichita)$^{\alpha}$	USA	Collection of Crop Research Division, U.S.D.A., Beltsville,
101	KU-366	- (April Bearded)$^{\alpha}$	UK	UK landrace, traditional variety, (spring),
102	KU-370	- (Renown)$^{\alpha}$	UK	Introduced in 1910, Squareheads Master x a Swedish Master, (winter),
103	KU-371	- (Wilhelmina)	UK	Introduced in 1910, Nethrelands: landrace x Squareheads Master, (winter),
104	KU-372	- (Little Joss)	UK	Introduced in 1910, Cambridge, UK: Squareheads Master x Ghirka (Russian), (winter),
105	KU-373	- (Victor)	UK	Introduced in 1910, Squareheads x Red King, (facultative),
106	KU-479	graecum Koern.	China	Suburbs of Chengtu, Szechwan,
107	KU-481	graecum Koern.	China	Suburbs of Chengan (Sian), Shensi,
108	KU-483	erythrospermum Koern.$^{\alpha}$	Tanzania	Collected in Tanzania,
109	KU-497	[local variety]$^{\alpha}$	India	Ootakamund, Nirgiris Hills, Tamil Nadu, a threshed sample was obtained from local seed shop.
110	KU-504	[local variety]$^{\alpha}$	China	concv. yunnanense (= T. yunnanense King), Awned, glabrous, white spike, red grain.
111	KU-601	- (Gifu Wase Komugi)$^{\alpha}$	Japan	Japanese landrace, traditional variety.
112	KU-1002	[local variety]$^{\alpha}$	Spain	Canada del Hoyo, Cuenca,
113	KU-1005	[local variety]$^{\alpha}$	Spain	Alba de los Cardanos, Palencia,
114	KU-1011	[local variety]$^{\alpha}$	Spain	Pedrosa del Rey, Leon,
115	KU-1049	[local variety]$^{\alpha}$	Spain	Coto de Somiedo, Oviedo,
116	KU-1143	[local variety]$^{\alpha}$	Spain	Castro-Cillorigo, Santander,
117	KU-1215	ferrugineum Alef. (Akita Zairai)$^{\alpha}$	Japan	Collection of National Agr. Exp. Sta., Kantoo-Toozan, Koonosu, (Endemic cultivar of Japan),
118	KU-1230	creticum Mazz. (Fukoku)$^{\alpha}$	Japan	Collection of National Agr. Exp. Sta., Kantoo-Toozan, Koonosu, (Endemic cultivar of Japan),
119	KU-1279	lutescens Alef. (Murasaki Aka)$^{\alpha}$	Japan	Collection of National Agr. Exp. Sta., Kantoo-Toozan, Koonosu, (Endemic cultivar of Japan),
120	KU-1302	[local variety]$^{\alpha}$	Greece	Near Petlarona, Macedonia,

Supplementary Table S1. (Continued).

Variety No.	ID	Variety (Cultivar) name / Species	Country	Locarity
121	KU-1347	[local variety]$^{\alpha}$	Greece	12 km S from Patra to Kalavrita, Peloponnesos,
122	KU-1392	[local variety]$^{\alpha}$	Romania	Dimbul, Jud. Cluj,
123	KU-1394	[local variety]$^{\alpha}$	Romania	Liviu Rebreanu, Jud. Bistrita-Nasaud,
124	KU-1421	[local variety]$^{\alpha}$	Romania	Julesti, Jud. Oradea,
125	KU-1424	[local variety]$^{\alpha}$	Romania	Bixad Cooperative Farm. Bixad, Jud. Satu Mare,
126	KU-1521	lutescens Alef.$^{\alpha}$	USSR	Baku, Azerbaijan,
127	KU-1527	milturum Alef.$^{\alpha}$	USSR	Erevan, Armenia,
128	KU-1644	graecum Koern.$^{\alpha}$	USSR	34 km NW of Erevan, Bjurakan, Armenia,
129	KU-1668	lutescens Alef.	USSR	Suburbs of Tibilisi, Georgia,
130	KU-1697	lutescens Alef.$^{\alpha}$	USSR	5 km S of Sagarenzho (Tibilisi - Telavi), Georgia,
131	KU-1797	lutescens Alef.$^{\alpha}$	USSR	7 km NE of Borzhormi (Tibilisi - Bakuriani), Georgia,
132	KU-3006	graecum Koern.$^{\alpha}$	Pakistan	Hudda (Suburbs of Quetta),
133	KU-3010	lut-inflatum Vav.$^{\alpha}$	Pakistan	Suburbs of Quetta,
134	KU-3037	subgraecum Vav.$^{\alpha}$	Pakistan	Suburbs of Chaman,
135	KU-3045	meridionale Koern.$^{\alpha}$	Afghanistan	Suburbs of Kandahar,
136	KU-3054	hostianum-compactoides Goekg.$^{\alpha}$	Afghanistan	Jaldak,
137	KU-3062	subturcicum Vav.	Afghanistan	Kabul,
138	KU-3083	erythroleucon Koern.$^{\alpha}$	Afghanistan	17 km N of Doshi, (Doshi - Pul-i-Khumri),
139	KU-3089	ferrugineum-compactoides Kob.$^{\alpha}$	Afghanistan	Maimana,
140	KU-3097	erythrospermum Koern.	Iran	10 km S of Torbat-jam (Herat - Meshhad),
141	KU-3098	subferrugineum-inflatum Palm.$^{\alpha}$	Iran	70 km S of Meshhad (Herat - Meshhad),
142	KU-3121	khorossanicum Vav.$^{\alpha}$	Iran	8 km SW of Isfahan (Isfahan - Shohr - Kord),
143	KU-3126	nigromeridionale Goekg.$^{\alpha}$	Iran	43 km SW of Isfahan (Isfahan - Shohr - Kord),
144	KU-3136	lut-inflatum Vav.$^{\alpha}$	Iran	40 km NE of Sefied-dasht (Shar - Kord - Isfahan),

Supplementary Table S1. (Continued).

Variety No.	ID	Variety (Cultivar) name / Species	Country	Locarity
145	KU-3162	new variety (echinodes-inflatum Vav. et Kob. with lax ear)$^{\alpha}$	Iran	Tehran - Karaj,
146	KU-3184	erythrospermum-compactoides Kob.	Iran	5 km W of Behshahr (Sari - Behshahr),
147	KU-3189	erythrospermum-compactoides Kob.$^{\alpha}$	Iran	Suburbs of Gorgan (Behshahr - Gorgan),
148	KU-3202	ferrugineum Alef.$^{\alpha}$	Iran	15 km ENE of Tehran (Tehran - Sari),
149	KU-3232	erythrospermum Koern.$^{\alpha}$	Iran	52 km E of Chalus (Babulsar - Chalus),
150	KU-3236	erythrospermum-compactoides Kob.	Iran	42 km SW of Astara (Astara - Ardabil),
151	KU-3274	meridionale Koern.$^{\alpha}$	Iran	Suburbs of Tabriz,
152	KU-3289	meridionale Koern.$^{\alpha}$	Iran	6 km N of Rezaiye (Rezaiye - Khoy),
153	KU-3299	barbarossa Alef.$^{\alpha}$	Pakistan	Taxilld, Karakoram,
154	KU-3351	ferrugineum Alef.$^{\alpha}$	Pakistan	Nomal, Karakoram,
155	KU-3752	nigroferrugineum Jakuschk.	Egypt	Saqqara (40 km S of Cairo),
156	KU-3777	erythrospermum Koern.$^{\alpha}$	Jordan	2 km S of the bordar (Damascus - Amman),
157	KU-3778	milturum Alef.$^{\alpha}$	Lebanon	20 km S of Saida,
158	KU-3780	alborubrum Koern.$^{\alpha}$	Syria	Suburbs of Kamicliye,
159	KU-3784	albocaesium Goekg.$^{\alpha}$	Turkey	10 km SW of Kirikhan (Aleppo - Adana),
160	KU-3789	chiovendae Vav. et Jakubz.$^{\alpha}$	Turkey	10 km W of Bursa (Bursa - Bandirma),
161	KU-3801	new variety (albocaesium Goekg. with brown grains)	Turkey	1 km SW of Edinak (Bandirma - Goenen),
162	KU-3806	alborubrum Koern.$^{\alpha}$	Turkey	23 km E of Adapazari (Izmit - Bolu),
163	KU-3818	turcicum-compactoides Zhuk.$^{\alpha}$	Turkey	12 km NW of Yerkoy (Yozgat - Cerikli),
164	KU-3834	erythrospermum-compactoides Kob.$^{\alpha}$	Turkey	24 km E of Ayas (Ankara - Ayas),
165	KU-3851	aleschkertianum Goekg.$^{\alpha}$	Turkey	13 km NE of Ankara,
166	KU-3857	ferrugineum Alef.$^{\alpha}$	Turkey	7 km E of Izmit (Bolu - Izmit),
167	KU-3860	ferrugineum Alef.$^{\alpha}$	Turkey	Suburbs of Serike,
168	KU-3868	creticum Mazz.$^{\alpha}$	Italy	79 km SE of Rome (Rome - Naples),

Supplementary Table S1. (Continued).

Variety No.	ID	Variety (Cultivar) name / Species	Country	Locarity
169	KU-4703	suberythroleucon Koern.$^{\alpha}$	Nepal	Katmandu, Nepal by Dr. Nakao,
170	KU-4714	milturum Alef.$^{\alpha}$	Nepal	Gho, Nepal by Dr. Nakao,
171	KU-4734	erythrospermum Koern.$^{\alpha}$	Nepal	Macha Khola, Nepal by Dr. Nakao,
172	KU-4759	erythrospermum Koern.$^{\alpha}$	Nepal	Ulleri, Nepal by Dr. Nakao,
173	KU-4769	delfii Koern.$^{\alpha}$	Nepal	Thonje, Nepal by Dr. Nakao,
174	KU-4783	erythrospermum Koern.	Nepal	Terai side, Nepal by Dr. Nakao,
175	KU-7001	nigromelanopogon Goekg.$^{\alpha}$	Bhutan	Collected in Bhutan by Dr. Nakao,
176	KU-7041	lutescens Alef.$^{\alpha}$	Bhutan	Collected in Bhutan by Dr. Nakao,
177	KU-7113	milturum Alef.$^{\alpha}$	Bhutan	Collected in Bhutan by Dr. Nakao,
178	KU-7180	subpseudomeridionale Vav.$^{\alpha}$	Bhutan	Collected in Bhutan by Dr. Nakao,
179	KU-7356	graecum Koern.$^{\alpha}$	Ethiopia	123 km SW of Addis Ababa toward Walisa,
180	KU-7379	erythroleucon Koern.$^{\alpha}$	Ethiopia	Shebe, 50 km SW of Jimma,
181	KU-7406	erythroleucon Koern.$^{\alpha}$	Ethiopia	Adaba market, origin Fatima (20 km to N),
182	KU-7437	suberythroleucon Koern.	Afghanistan	1 km E of Armalik, Herat,
183	KU-7459	ferrugineum Alef.$^{\alpha}$	Afghanistan	1.5 km W of Qala Nau, Badghis,
184	KU-7480	subhostianum Vav.$^{\alpha}$	Afghanistan	Junction of Farah rud and Khorbordar River, Ghor,
185	KU-7624	subferrugineum Vav.	Afghanistan	15 km W of Bamian (Panjao - Bamian), Bamiyan,
186	KU-7653	barbarossa Alef.$^{\alpha}$	Afghanistan	5 km W of Tash-Kurghan, near Khulm, Samangan,
187	KU-7669	subhostianum Vav.$^{\alpha}$	Afghanistan	16 km W of Kandahar, Kandahar,
188	KU-9431	graecum Koern.$^{\alpha}$	Ethiopia	Mt. Entoto, Addis Ababa,
189	KU-9460	erythrospermum Koern.$^{\alpha}$	Ethiopia	10 km from Jijiga to Harrar,
190	KU-9797	ferrugineum Alef.	Ethiopia	90 km N of Assella,
191	KU-9820	lutescens Alef.$^{\alpha}$	Ethiopia	58 km NW of Addis Ababa (Gozu market),
192	KU-9867	ferrugineum Alef.$^{\alpha}$	Ethiopia	Kurfa Chellay (Harar),

Supplementary Table S1. (Continued).

Variety No.	ID	Variety (Cultivar) name / Species	Country	Locarity
193	KU-10001	erythrospermum Koern.$^{\alpha}$	Iraq	Baghdad,
194	KU-10393	erythrospermum Koern.	Iran	65.7 km from Mianeh to Tehran,
195	KU-10439	graecum Koern.	Iran	34.6 km NE from Hamadan to Qazvin,
196	KU-10480	erythrospermum Koern.$^{\alpha}$	Iran	37.7 km W from Sanandaj to Dezh Shahpur,
197	KU-10510	erythrospermum Koern.$^{\alpha}$	Iran	5.3 km SE from Karand to Shahabad,
198	KU-11201	erythrospermum Koern.$^{\alpha}$	Afghanistan	Bazar of Kabul,
199	KU-11214	erythrospermum Koern.$^{\alpha}$	Afghanistan	37 km E of Taluquan, near Kalafgan, Takhar,
200	KU-11240A	erythrospermum Koern.$^{\alpha}$	Afghanistan	Bamdara, 13 km N of Robobi to Shewa, Badakhshan,
201	KU-11351	ferrugineum Alef.$^{\alpha}$	Romania	Singeorz Bai, Jud. Bistrita-Nasaud,
202	KU-11702	[local variety]$^{\alpha}$	Greece	9.3 km W from Irakrion airport to Metochi Kapetanaki, Crete,
203	KU-11809	[local variety]$^{\alpha}$	Greece	Eastern outside of Apolakia, Rhodes,
204	KU-11829	[local variety]$^{\alpha}$	Greece	Skala Eressou, Lesbos,
205	KU-13501	[local variety]$^{\alpha}$	China	Southern suberb of Taoping, Sichuan,
206	KU-13506	[local variety]	China	Lhasa - Qushui, Xizan,
207	KU-13546	[local variety]$^{\alpha}$	China	South of Nedong, Xizan,
208	KU-13631	[local variety]$^{\alpha}$	China	South of Qiongjie, Xizan,
209	KU-13662	[local variety]$^{\alpha}$	China	Lahsa - Yangbajain, Xizan,
210	KU-13708	[local variety]$^{\alpha}$	China	Lhasa - Mozhugongka, Xizan,
211	KU-13807	[local variety]$^{\alpha}$	China	West of Xigaze, Xizan,
212	KU-13891	[local variety]$^{\alpha}$	China	27 km west from Gongbogyamda on the way to Lhasa, Xizan,
213	KU-152	Triticum compactum$^{\alpha}$	China	
214	KU-1208	Triticum compactum$^{\alpha}$	Japan	
215	KU-3063	Triticum compactum$^{\alpha}$	Afganistan	
216	KU-3242	Triticum compactum$^{\alpha}$	Iran	

Supplementary Table S1. (Continued).

Variety No.	ID	Variety (Cultivar) name / Species	Country	Locarity
217	KU-7350	Triticum compactum[α]	Turkey	
218	KU-161	Triticum sphaerocuccum	-	
219	KU-162-2	Triticum sphaerocuccum[α]	Pakistan	
220	KU-3004	Triticum sphaerocuccum	Pakistan	
221		Opata M85[α]	Mexico	
222		Timstein[α]	Australia	
223		Hope[α]	UK	
224		Cheyenne[α]	Germany	
225		Zenkoji Komugi[α]	Japan	
226		Sumai 3	China	
227		KS831957[α]	USA	
228		U24[α]	China	
229		Minamino Komugi[α]	Japan	
230		Chogokuwase	Japan	
231		Akimakigata Abukumawase[α]	Japan	
232		Nishikaze Komugi[α]	Japan	
233		Gamenya	Australia	
234		Akadaruma (S08 AK self)[α]	Japan	

[α] Varieties evaluted in both 2012-2013 and 2013-2014 growing season.

Supplementary Table S2. Traits related to RCs accumulation of each variety evaluated in this study.

Variety No.	Grain RCs concentration		Shoot RCs concentration		Residual (soil ExK - grain)		Residual (soil ExK - shoot)		RCs ratio of grain to shoot		TF[α]	
	2012-2013	2013-2014	2012-2013	2013-2014	2012-2013	2013-2014	2012-2013	2013-2014	2012-2013	2013-2014	2012-2013	2013-2014
1	25.9	35.3	28.6	61.1	-5.2	2.1	-46.7	-22.2	0.78	0.58	0.006	0.009
2	57.0	34.2	111.0	87.2	34.3	2.6	52.4	7.9	0.51	0.39	0.015	0.009
3	61.5	57.0	123.2	82.1	21.7	23.7	29.3	-0.9	0.51	0.69	0.014	0.017
4	102.6	29.7	170.0	48.2	52.6	0.9	55.8	-24.5	0.60	0.62	0.024	0.008
5	33.0	33.6	108.1	88.7	-4.6	-0.1	18.7	4.5	0.32	0.38	0.008	0.008
6	55.6	48.0	108.6	104.9	23.5	24.7	29.8	45.1	0.51	0.46	0.015	0.016
7	55.6	-[β]	153.7	-	24.1	-	76.1	-	0.37	-	0.013	-
8	10.3	26.3	33.0	68.6	-9.9	5.6	-20.3	15.1	0.33	0.38	0.002	0.007
9	46.1	-	144.8	-	2.9	-	44.5	-	0.31	-	0.010	-
10	14.1	15.3	29.2	45.1	-9.6	-12.0	-31.9	-24.0	0.47	0.34	0.004	0.004
11	43.1	56.7	104.4	166.7	10.9	14.8	26.6	63.1	0.40	0.34	0.010	0.015
12	24.2	40.0	63.8	104.5	-3.7	11.5	-6.1	32.4	0.37	0.38	0.006	0.011
13	48.8	-	122.9	-	18.7	-	48.3	-	0.40	-	0.011	-
14	18.8	36.9	55.4	162.0	-3.6	-3.6	-3.0	62.2	0.34	0.23	0.004	0.010
15	28.6	29.6	118.5	146.2	-11.7	-0.8	23.6	69.6	0.24	0.20	0.007	0.007
16	21.9	69.3	52.4	163.3	-8.9	39.9	-22.9	89.0	0.39	0.42	0.005	0.017
17	51.0	-	73.9	-	22.1	-	1.5	-	0.68	-	0.012	-
18	48.5	-	160.6	-	-9.4	-	31.2	-	0.30	-	0.013	-
19	23.5	66.7	75.2	143.4	6.4	28.4	28.7	48.2	0.30	0.47	0.006	0.018
20	20.8	27.7	59.9	98.8	-9.0	1.5	-14.3	32.1	0.35	0.28	0.006	0.008
21	57.3	-	124.0	-	9.8	-	15.2	-	0.44	-	0.015	-
22	60.6	33.2	91.6	64.5	28.1	1.7	11.9	-14.6	0.68	0.51	0.014	0.011
23	13.6	71.4	24.6	109.8	-4.7	36.4	-24.5	22.4	0.50	0.65	0.004	0.014
24	14.3	20.6	55.4	82.4	-9.4	-6.7	-5.8	13.0	0.27	0.25	0.004	0.007
25	37.1	46.2	97.7	160.6	4.1	14.9	17.6	82.2	0.37	0.29	0.009	0.012
26	44.4	32.8	118.6	88.4	6.7	1.3	29.1	9.1	0.38	0.37	0.013	0.008
27	19.4	-	50.3	-	-1.1	-	-3.7	-	0.40	-	0.005	-

Supplementary Table S2. (Continued).

Variety No.	Grain RCs concentration		Shoot RCs concentration		Residual (soil ExK - grain)		Residual (soil ExK - shoot)		RCs ratio of grain to shoot		TF[α]	
	2012-2013	2013-2014	2012-2013	2013-2014	2012-2013	2013-2014	2012-2013	2013-2014	2012-2013	2013-2014	2012-2013	2013-2014
28	55.1	46.7	239.3	169.5	21.8	13.9	157.9	87.4	0.24	0.28	0.012	0.009
29	25.3	44.9	71.0	137.0	-4.3	7.9	-2.6	44.9	0.36	0.33	0.006	0.013
30	40.7	60.9	90.9	177.5	0.8	26.9	-2.0	92.6	0.41	0.34	0.009	0.013
31	41.4	41.6	86.8	106.1	21.0	12.1	32.7	31.9	0.50	0.39	0.010	0.011
32	31.9	34.9	78.3	101.6	-8.1	5.2	-16.7	26.5	0.39	0.34	0.009	0.009
33	32.9	70.3	68.3	139.3	-4.6	31.6	-20.1	43.3	0.44	0.50	0.009	0.015
34	63.2	47.2	183.5	126.0	35.4	-3.7	114.3	1.8	0.29	0.37	0.013	0.011
35	18.3	32.5	58.6	105.0	-0.9	5.4	7.4	36.4	0.31	0.31	0.004	0.007
36	33.7	24.9	111.0	70.1	0.6	-7.0	30.6	-10.0	0.30	0.36	0.009	0.007
37	24.2	47.8	45.0	147.0	-0.3	22.7	-17.7	83.1	0.54	0.33	0.006	0.016
38	44.4	54.2	82.1	141.4	6.5	15.0	-7.8	44.2	0.54	0.38	0.011	0.014
39	26.8	29.7	67.0	66.8	7.5	-9.6	15.6	-30.5	0.42	0.44	0.007	0.007
40	13.8	18.7	33.9	87.8	-6.7	-19.4	-19.2	-6.6	0.42	0.21	0.003	0.005
41	49.2	34.6	103.9	88.2	13.3	-1.5	17.7	-1.9	0.47	0.39	0.013	0.008
42	21.8	42.3	45.4	135.6	-1.5	2.6	-14.7	37.5	0.46	0.31	0.006	0.012
43	51.7	34.0	71.2	67.5	17.1	5.3	-12.9	-4.9	0.71	0.50	0.014	0.006
44	37.0	45.5	92.4	179.4	-5.9	11.3	-7.5	94.0	0.40	0.25	0.009	0.012
45	37.8	27.4	86.0	58.0	11.1	-7.0	18.6	-28.0	0.46	0.47	0.009	0.007
46	47.9	59.6	116.2	108.5	15.1	17.8	36.0	5.4	0.42	0.55	0.011	0.012
47	25.2	34.8	55.1	101.4	-3.2	-2.8	-15.2	8.0	0.45	0.34	0.006	0.013
48	31.8	47.5	105.2	161.1	-7.3	11.5	13.2	71.5	0.32	0.29	0.008	0.015
49	44.4	29.9	94.2	67.0	-1.6	-5.1	-12.6	-19.9	0.47	0.45	0.012	0.007
50	34.0	-	79.8	-	5.3	-	8.0	-	0.43	-	0.008	-
51	30.0	29.6	63.3	99.7	-5.0	1.8	-20.5	29.3	0.49	0.30	0.007	0.007
52	15.2	29.2	37.6	118.0	-25.7	-2.6	-57.9	38.1	0.45	0.25	0.004	0.008
53	29.8	26.6	78.1	82.0	-7.6	-5.5	-10.1	1.5	0.32	0.32	0.007	0.007
54	94.9	56.2	120.2	108.1	51.8	12.8	20.6	1.2	0.69	0.52	0.027	0.015

Supplementary Table S2. (Continued).

Variety No.	Grain RCs concentration		Shoot RCs concentration		Residual (soil ExK - grain)		Residual (soil ExK - shoot)		RCs ratio of grain to shoot		TF[α]	
	2012-2013	2013-2014	2012-2013	2013-2014	2012-2013	2013-2014	2012-2013	2013-2014	2012-2013	2013-2014	2012-2013	2013-2014
55	18.1	25.5	79.2	97.5	-17.7	-17.2	-6.4	-7.9	0.23	0.26	0.004	0.007
56	38.5	29.8	139.4	129.3	3.7	-4.1	55.1	44.4	0.26	0.23	0.010	0.008
57	23.9	-	95.1	-	-1.2	-	30.8	-	0.25	-	0.007	-
58	21.1	43.1	55.9	96.7	-11.6	10.2	-23.5	14.5	0.35	0.45	0.005	0.012
59	29.3	34.2	71.3	118.0	-5.8	0.7	-14.0	34.4	0.41	0.29	0.007	0.012
60	41.5	-	130.5	-	-8.5	-	19.8	-	0.28	-	0.011	-
61	26.5	27.0	80.9	74.8	-2.4	-1.6	8.8	2.2	0.33	0.36	0.006	0.007
62	28.9	36.1	82.9	126.0	-10.9	7.0	-10.3	52.5	0.36	0.29	0.008	0.009
63	39.5	22.0	91.4	65.2	-8.0	-6.1	-17.9	-5.8	0.41	0.34	0.011	0.006
64	57.4	21.3	160.7	62.2	19.6	-5.0	71.2	-4.7	0.37	0.34	0.013	0.006
65	26.6	31.8	67.6	94.4	-4.2	-11.0	-8.3	-11.1	0.39	0.34	0.008	0.008
66	53.3	40.9	181.6	133.9	22.7	10.3	105.6	57.1	0.29	0.31	0.013	0.012
67	64.1	-	206.3	-	22.7	-	108.9	-	0.31	-	0.014	-
68	68.0	22.9	145.3	58.4	24.8	-0.1	44.8	-0.6	0.46	0.39	0.017	0.007
69	37.6	30.4	88.5	80.9	2.6	-7.2	3.8	-12.4	0.43	0.38	0.008	0.008
70	34.9	32.7	102.2	88.0	2.6	0.2	23.0	6.6	0.34	0.37	0.008	0.008
71	21.9	-	57.7	-	-9.7	-	-18.2	-	0.38	-	0.006	-
72	33.8	37.4	91.0	73.3	7.7	-4.2	25.5	-29.1	0.35	0.51	0.008	0.008
73	19.1	26.3	54.8	82.7	-6.8	-12.3	-11.1	-13.0	0.33	0.32	0.005	0.007
74	57.0	23.6	153.8	78.9	24.5	-7.7	74.0	0.4	0.36	0.30	0.015	0.006
75	43.7	26.4	102.1	71.2	11.2	0.7	23.5	5.7	0.48	0.37	0.012	0.008
76	52.1	69.1	189.6	266.2	6.7	32.3	84.1	174.6	0.28	0.26	0.011	0.018
77	15.1	21.7	33.3	75.3	-4.3	-10.2	-18.2	-4.8	0.46	0.29	0.004	0.007
78	20.9	31.3	46.2	91.8	-7.5	4.2	-25.0	22.9	0.45	0.34	0.005	0.010
79	20.4	44.3	53.0	76.2	-2.7	10.4	-6.9	-8.7	0.36	0.58	0.004	0.014
80	13.3	-	43.5	-	-7.8	-	-11.4	-	0.30	-	0.003	-
81	29.4	31.5	109.0	110.9	-10.6	2.4	15.8	37.7	0.27	0.28	0.007	0.008

Supplementary Table S2. (Continued).

Variety No.	Grain RCs concentration		Shoot RCs concentration		Residual (soil ExK - grain)		Residual (soil ExK - shoot)		RCs ratio of grain to shoot		TF[α]	
	2012-2013	2013-2014	2012-2013	2013-2014	2012-2013	2013-2014	2012-2013	2013-2014	2012-2013	2013-2014	2012-2013	2013-2014
82	29.1	20.8	87.3	79.6	7.4	-7.2	30.9	8.7	0.34	0.26	0.008	0.005
83	57.2	24.7	134.7	79.4	17.3	-14.3	39.9	-17.5	0.44	0.31	0.013	0.008
84	44.9	17.7	135.4	94.4	1.2	-12.1	33.0	19.3	0.34	0.19	0.011	0.005
85	36.6	30.5	100.0	83.0	-1.0	3.2	9.9	13.6	0.36	0.37	0.009	0.010
86	48.1	45.1	162.9	146.2	5.9	13.5	65.3	66.9	0.30	0.31	0.012	0.013
87	20.1	30.2	59.9	86.0	-11.5	-5.0	-17.6	-1.5	0.33	0.35	0.005	0.009
88	14.9	56.2	51.4	160.9	-6.0	19.4	-3.6	69.6	0.28	0.35	0.005	0.015
89	19.2	47.2	48.5	148.5	-1.1	9.8	-5.0	55.5	0.38	0.32	0.005	0.011
90	14.7	37.5	36.7	90.8	-9.0	13.1	-23.9	28.5	0.40	0.41	0.004	0.012
91	32.2	47.9	78.7	144.6	6.5	7.2	13.3	44.2	0.41	0.33	0.009	0.014
92	35.5	22.0	85.6	68.8	-7.6	-5.0	-14.4	0.2	0.38	0.32	0.008	0.006
93	24.7	-	62.4	-	-1.0	-	-3.0	-	0.39	-	0.007	-
94	14.6	43.0	34.3	125.9	-1.8	4.4	-10.7	30.1	0.42	0.34	0.004	0.011
95	26.1	33.6	57.5	96.7	1.5	3.5	-5.7	20.9	0.46	0.35	0.006	0.009
96	53.9	36.5	112.5	114.2	5.5	0.9	1.1	25.5	0.46	0.32	0.016	0.011
97	22.0	40.6	73.0	112.3	-25.7	-7.8	-36.8	-6.1	0.29	0.36	0.006	0.010
98	38.4	42.3	58.0	84.9	8.0	3.3	-17.5	-11.7	0.65	0.50	0.010	0.011
99	104.6	49.2	204.8	113.8	38.3	11.8	61.9	20.9	0.47	0.43	0.024	0.014
100	28.9	36.7	50.8	74.4	-6.0	-2.8	-33.2	-23.4	0.59	0.49	0.007	0.011
101	55.3	43.7	104.4	92.2	25.6	1.6	30.4	-11.7	0.53	0.47	0.013	0.012
102	61.7	25.6	165.5	61.6	15.9	-0.7	59.7	-5.2	0.34	0.42	0.014	0.007
103	21.0	-	65.2	-	-13.3	-	3.9	-	0.37	-	0.005	-
104	28.2	-	88.6	-	-12.0	-	-5.7	-	0.26	-	0.006	-
105	31.4	-	96.4	-	5.1	-	30.5	-	0.28	-	0.006	-
106	35.6	-	97.0	-	5.7	-	22.9	-	0.37	-	0.008	-
107	38.0	21.4	93.2	66.7	11.4	-6.9	25.7	-4.8	0.41	0.32	0.009	0.006
108	28.8	16.6	83.5	61.0	-7.3	-8.9	-3.0	-4.0	0.30	0.27	0.008	0.004

Supplementary Table S2. (Continued).

Variety No.	Grain RCs concentration		Shoot RCs concentration		Residual (soil ExK - grain)		Residual (soil ExK - shoot)		RCs ratio of grain to shoot		TF[α]	
	2012-2013	2013-2014	2012-2013	2013-2014	2012-2013	2013-2014	2012-2013	2013-2014	2012-2013	2013-2014	2012-2013	2013-2014
109	15.5	33.2	62.6	108.7	-8.0	-2.1	1.8	20.9	0.27	0.31	0.004	0.009
110	54.7	23.3	154.8	83.3	13.5	-4.2	58.0	13.5	0.36	0.28	0.014	0.006
111	45.7	50.6	119.1	130.3	12.3	28.2	37.8	72.8	0.37	0.39	0.011	0.014
112	60.2	38.7	98.2	67.9	17.7	0.0	-1.5	-28.1	0.59	0.57	0.016	0.011
113	40.6	24.3	68.5	37.6	7.4	1.8	-12.8	-20.2	0.60	0.65	0.009	0.006
114	51.1	42.6	90.7	59.9	19.3	10.5	12.8	-20.9	0.58	0.71	0.013	0.014
115	50.3	19.3	119.0	62.8	-1.8	-11.5	0.4	-14.5	0.41	0.31	0.013	0.006
116	70.6	46.1	163.1	96.1	19.9	9.4	47.9	4.7	0.45	0.48	0.019	0.011
117	53.3	37.6	105.3	69.2	25.1	8.7	34.6	-4.1	0.48	0.54	0.013	0.010
118	16.9	23.6	40.5	73.1	-5.7	-16.7	-17.5	-25.7	0.39	0.32	0.005	0.006
119	63.9	16.9	162.5	41.5	31.5	-7.6	83.1	-21.0	0.41	0.41	0.018	0.005
120	42.7	49.1	89.6	126.2	14.4	12.7	18.5	35.6	0.45	0.39	0.009	0.011
121	70.1	64.6	93.9	92.7	26.0	21.6	-8.9	-12.8	0.69	0.70	0.017	0.018
122	53.3	54.1	106.4	130.6	18.1	19.3	21.2	43.7	0.47	0.41	0.012	0.014
123	40.3	42.9	72.3	86.0	3.2	12.9	-14.9	10.1	0.54	0.50	0.010	0.009
124	52.9	24.1	96.8	43.2	14.3	-5.4	4.8	-31.5	0.54	0.56	0.012	0.006
125	59.1	32.9	169.8	116.8	16.9	-10.9	70.5	8.8	0.37	0.28	0.014	0.008
126	66.8	33.0	172.0	88.6	17.2	-2.5	58.3	0.1	0.39	0.37	0.017	0.008
127	39.8	37.2	81.5	72.3	-12.4	2.2	-37.1	-14.5	0.48	0.51	0.010	0.009
128	41.0	39.8	84.8	54.9	8.6	12.4	5.4	-14.4	0.45	0.72	0.009	0.010
129	73.4	-	153.2	-	42.8	-	77.3	-	0.49	-	0.019	-
130	17.6	37.5	58.5	112.0	-10.2	2.2	-10.9	23.7	0.30	0.34	0.004	0.006
131	62.2	15.3	134.7	57.1	21.6	-8.4	38.9	-3.5	0.46	0.27	0.013	0.004
132	22.4	62.6	62.1	106.3	0.2	34.8	5.0	36.0	0.38	0.59	0.006	0.021
133	21.3	34.3	51.3	100.3	-12.1	-17.6	-30.2	-26.0	0.41	0.34	0.006	0.011
134	21.8	27.7	57.4	80.1	-11.5	-2.6	-22.9	3.8	0.36	0.35	0.006	0.006
135	33.2	51.1	79.5	91.8	-0.8	15.2	-2.2	2.4	0.44	0.56	0.007	0.013

Supplementary Table S2. (Continued).

Variety No.	Grain RCs concentration		Shoot RCs concentration		Residual (soil ExK - grain)		Residual (soil ExK - shoot)		RCs ratio of grain to shoot		TF[α]	
	2012-2013	2013-2014	2012-2013	2013-2014	2012-2013	2013-2014	2012-2013	2013-2014	2012-2013	2013-2014	2012-2013	2013-2014
136	45.5	49.1	113.7	88.8	9.4	14.0	27.1	0.9	0.40	0.55	0.012	0.013
137	51.1	-	113.4	-	-2.1	-	-6.9	-	0.45	-	0.013	-
138	45.6	23.8	81.3	35.7	-12.4	-8.6	-48.5	-45.6	0.55	0.67	0.011	0.008
139	38.1	15.1	66.4	29.9	7.1	-13.8	-9.0	-43.0	0.49	0.50	0.008	0.005
140	51.7	-	114.5	-	4.5	-	5.3	-	0.44	-	0.011	-
141	36.8	40.4	76.4	86.7	-0.3	11.9	-12.5	14.7	0.49	0.47	0.008	0.012
142	41.5	25.9	98.8	51.0	9.4	1.6	19.9	-11.3	0.42	0.51	0.008	0.008
143	57.5	19.1	104.1	40.0	1.1	-9.4	-20.5	-32.3	0.49	0.48	0.016	0.004
144	27.6	39.5	82.4	76.9	-8.9	2.4	-4.3	-15.2	0.28	0.51	0.007	0.011
145	28.2	47.0	74.8	119.0	3.2	11.4	10.9	30.2	0.37	0.39	0.006	0.012
146	54.7	-	67.7	-	12.0	-	-31.7	-	0.80	-	0.013	-
147	21.1	55.5	46.3	85.9	-6.7	26.3	-23.6	12.0	0.49	0.65	0.006	0.012
148	26.0	37.9	49.7	92.4	-7.0	-8.6	-30.9	-21.8	0.50	0.41	0.007	0.012
149	75.7	28.9	108.6	56.1	46.0	-1.1	34.9	-19.5	0.62	0.52	0.017	0.007
150	55.0	-	81.3	-	18.7	-	-6.3	-	0.67	-	0.013	-
151	49.8	35.7	65.1	47.4	-22.2	14.4	-43.8	-7.3	0.69	0.75	0.013	0.011
152	69.1	34.7	134.8	45.6	23.3	6.1	29.0	-26.7	0.51	0.76	0.016	0.011
153	45.7	51.0	106.2	122.1	11.6	13.8	26.7	29.7	0.33	0.42	0.011	0.012
154	38.0	51.1	76.7	141.2	9.4	10.3	5.0	40.4	0.48	0.36	0.009	0.012
155	20.5	-	112.4	-	-2.5	-	52.7	-	0.23	-	0.005	-
156	38.9	43.0	58.4	74.2	4.6	-0.4	-24.1	-32.7	0.62	0.58	0.009	0.012
157	23.3	23.8	86.2	82.6	-4.8	-8.1	17.0	2.7	0.33	0.29	0.006	0.006
158	59.3	17.6	247.0	64.7	23.7	-19.0	161.6	-26.4	0.27	0.27	0.013	0.005
159	34.9	55.5	82.0	131.4	10.5	20.2	19.5	43.3	0.42	0.42	0.008	0.016
160	11.0	35.0	29.7	88.1	-7.0	-3.8	-18.7	-8.2	0.38	0.40	0.003	0.009
161	31.4	-	65.5	-	6.2	-	1.4	-	0.47	-	0.007	-
162	44.5	25.3	95.4	75.7	0.0	-9.3	-7.7	-10.6	0.45	0.33	0.010	0.005

Supplementary Table S2. (Continued).

Variety No.	Grain RCs concentration		Shoot RCs concentration		Residual (soil ExK - grain)		Residual (soil ExK - shoot)		RCs ratio of grain to shoot		TF$^{\alpha}$	
	2012-2013	2013-2014	2012-2013	2013-2014	2012-2013	2013-2014	2012-2013	2013-2014	2012-2013	2013-2014	2012-2013	2013-2014
163	40.2	44.8	101.4	116.7	-2.8	10.4	1.2	30.7	0.40	0.38	0.011	0.012
164	76.8	64.7	145.7	87.6	11.7	19.8	3.0	-22.6	0.53	0.74	0.023	0.014
165	44.7	53.5	123.6	130.7	7.5	17.5	34.3	41.0	0.35	0.41	0.011	0.013
166	34.9	35.3	57.8	62.4	7.6	-1.5	-10.7	-29.2	0.59	0.57	0.008	0.007
167	58.8	60.9	113.4	101.8	-10.9	17.5	-34.4	-5.1	0.55	0.60	0.016	0.012
168	22.8	24.9	50.4	87.8	-5.4	-1.4	-20.1	21.1	0.42	0.28	0.005	0.006
169	65.5	55.5	125.5	106.6	31.0	11.0	42.1	-2.8	0.45	0.52	0.017	0.010
170	32.7	25.7	85.3	90.3	-3.5	-2.6	-1.7	19.0	0.39	0.28	0.008	0.007
171	50.4	22.7	107.0	59.2	19.4	-5.8	31.2	-12.7	0.47	0.38	0.013	0.006
172	36.0	35.9	60.0	56.8	-2.3	2.3	-30.3	-27.2	0.54	0.63	0.012	0.010
173	29.5	37.4	65.5	80.6	-14.0	3.3	-36.4	-4.6	0.45	0.46	0.008	0.010
174	27.8	-	69.0	-	-18.8	-	-38.4	-	0.38	-	0.006	-
175	53.7	38.2	122.8	95.4	17.0	-6.4	34.4	-14.3	0.44	0.40	0.012	0.011
176	34.3	30.5	74.4	65.3	-5.7	3.8	-20.2	-2.5	0.47	0.47	0.008	0.007
177	33.4	29.3	102.6	86.9	-5.3	3.7	10.8	21.8	0.32	0.34	0.008	0.010
178	35.6	40.8	89.6	73.9	15.5	8.6	36.3	-7.0	0.40	0.55	0.008	0.012
179	61.7	40.3	121.1	66.8	13.9	9.4	11.1	-11.0	0.53	0.60	0.013	0.014
180	37.4	15.1	77.3	39.8	10.3	-8.9	8.8	-21.4	0.51	0.38	0.009	0.005
181	57.8	40.1	84.3	91.6	19.9	1.3	-6.3	-4.7	0.68	0.44	0.013	0.011
182	76.0	-	168.1	-	34.4	-	70.4	-	0.46	-	0.018	-
183	33.4	26.9	57.7	54.3	1.2	5.0	-21.5	-2.1	0.58	0.49	0.009	0.006
184	37.0	46.6	69.8	70.5	-1.8	20.3	-22.2	3.7	0.50	0.66	0.009	0.015
185	30.1	-	110.7	-	2.7	-	42.2	-	0.24	-	0.007	-
186	92.9	55.7	119.4	97.1	15.7	21.6	-44.7	11.7	0.72	0.57	0.022	0.016
187	38.4	30.7	101.2	83.2	4.3	-18.2	18.1	-36.6	0.37	0.37	0.009	0.007
188	65.4	26.2	66.3	50.6	26.8	-14.0	-25.4	-49.0	0.86	0.52	0.016	0.008
189	49.0	51.8	88.0	99.2	-12.5	21.5	-48.3	23.1	0.55	0.52	0.012	0.014

Supplementary Table S2. (Continued).

Variety No.	Grain RCs concentration		Shoot RCs concentration		Residual (soil ExK - grain)		Residual (soil ExK - shoot)		RCs ratio of grain to shoot		TF[α]	
	2012-2013	2013-2014	2012-2013	2013-2014	2012-2013	2013-2014	2012-2013	2013-2014	2012-2013	2013-2014	2012-2013	2013-2014
190	57.7	-	96.7	-	14.1	-	-4.8	-	0.57	-	0.015	-
191	28.1	24.6	54.2	46.0	-15.5	2.3	-47.7	-11.3	0.52	0.54	0.008	0.005
192	37.2	51.6	56.1	115.3	-12.8	10.9	-57.0	14.8	0.64	0.45	0.011	0.013
193	82.5	26.6	127.2	44.0	34.0	3.0	15.6	-16.4	0.65	0.61	0.021	0.006
194	37.8	39.7	122.7	84.7	8.6	3.5	49.6	-5.4	0.35	0.47	0.008	0.011
195	44.7	-	113.3	-	-4.7	-	1.4	-	0.39	-	0.012	-
196	45.7	30.7	105.5	90.6	2.5	-5.4	4.3	0.7	0.43	0.34	0.010	0.009
197	51.1	35.1	89.7	82.3	-1.7	-1.9	-29.6	-9.7	0.57	0.43	0.014	0.012
198	86.3	19.1	135.1	47.1	29.7	-9.8	8.9	-25.9	0.64	0.40	0.022	0.007
199	39.5	58.6	73.1	120.0	8.8	18.9	-2.8	21.6	0.54	0.49	0.011	0.018
200	43.6	44.3	51.7	101.7	10.7	0.2	-28.2	-6.7	0.82	0.44	0.010	0.016
201	34.6	49.4	69.2	66.6	-0.5	17.0	-15.4	-14.6	0.51	0.74	0.008	0.013
202	39.5	46.4	90.6	84.1	10.7	-9.6	19.2	-51.6	0.43	0.55	0.009	0.015
203	36.0	52.5	69.0	103.8	7.8	11.4	-1.2	2.2	0.59	0.51	0.009	0.013
204	56.5	36.2	82.4	66.0	15.8	3.0	-11.9	-17.1	0.58	0.55	0.016	0.010
205	21.5	36.9	67.6	115.9	-9.5	-2.8	-9.1	18.0	0.32	0.32	0.006	0.013
206	45.8	-	106.1	-	7.5	-	15.2	-	0.42	-	0.011	-
207	85.4	54.8	285.8	139.3	35.2	8.3	171.1	25.7	0.30	0.39	0.021	0.014
208	21.4	39.6	95.3	98.9	-33.5	2.2	-28.5	6.1	0.24	0.40	0.006	0.009
209	38.4	45.8	100.1	107.1	-6.9	13.7	-1.4	26.5	0.42	0.43	0.010	0.016
210	27.2	38.1	97.3	102.7	2.8	3.3	34.5	15.8	0.31	0.37	0.006	0.007
211	45.5	33.4	140.0	96.9	-4.0	12.3	26.8	42.5	0.32	0.35	0.010	0.011
212	42.4	32.1	98.6	88.6	4.0	1.8	7.7	12.2	0.42	0.36	0.010	0.008
213	31.0	33.7	59.1	63.5	-4.5	-3.2	-26.2	-28.2	0.48	0.53	0.008	0.008
214	32.9	47.2	117.7	161.2	-10.2	9.4	16.7	67.3	0.27	0.29	0.008	0.013
215	57.0	23.8	97.0	39.3	32.8	-18.4	34.8	-64.7	0.64	0.61	0.013	0.006
216	30.0	46.6	113.1	88.8	-11.1	12.0	16.1	2.6	0.25	0.52	0.007	0.016

Supplementary Table S2. (Continued).

Variety No.	Grain RCs concentration		Shoot RCs concentration		Residual (soil ExK - grain)		Residual (soil ExK - shoot)		RCs ratio of grain to shoot		TF[α]	
	2012-2013	2013-2014	2012-2013	2013-2014	2012-2013	2013-2014	2012-2013	2013-2014	2012-2013	2013-2014	2012-2013	2013-2014
217	62.9	20.3	159.3	51.2	-3.4	-9.0	14.7	-22.8	0.42	0.40	0.016	0.005
218	27.5	-	78.8	-	2.2	-	14.2	-	0.36	-	0.006	-
219	14.3	29.8	86.3	91.7	-57.7	-3.0	-68.7	9.5	0.20	0.32	0.004	0.008
220	21.3	-	78.1	-	-6.6	-	8.5	-	0.27	-	0.004	-
221	19.2	15.4	46.3	36.6	-12.1	-19.1	-30.6	-49.7	0.45	0.42	0.005	0.004
222	30.3	37.7	129.8	84.8	-10.0	5.2	35.0	3.7	0.23	0.44	0.007	0.008
223	53.4	49.7	131.1	89.9	14.1	14.5	39.2	2.0	0.37	0.55	0.012	0.010
224	30.8	8.8	63.4	18.3	6.4	-13.9	0.8	-39.8	0.50	0.48	0.008	0.002
225	27.8	52.5	79.7	126.0	0.7	17.4	11.8	38.4	0.34	0.42	0.007	0.011
226	51.7	-	128.9	-	5.5	-	22.7	-	0.41	-	0.013	-
227	42.3	22.5	116.9	68.4	15.6	-15.0	49.6	-24.9	0.37	0.33	0.009	0.005
228	52.5	43.2	132.9	104.6	17.6	6.4	48.2	13.1	0.45	0.41	0.014	0.010
229	29.5	27.6	69.3	82.7	0.2	1.5	-3.7	16.5	0.43	0.33	0.007	0.010
230	23.1	-	60.1	-	-12.5	-	-26.1	-	0.40	-	0.006	-
231	18.8	37.5	64.9	121.8	-4.5	5.6	4.4	41.6	0.29	0.31	0.004	0.010
232	30.3	47.8	71.2	122.8	3.9	10.1	4.1	29.0	0.43	0.39	0.007	0.015
233	35.0	-	115.8	-	-6.2	-	18.4	-	0.30	-	0.007	-
234	15.3	36.4	41.0	95.2	-11.5	4.9	-26.0	15.9	0.37	0.38	0.004	0.008
average	39.9	37.2	94.3	92.9	4.2	3.5	9.3	8.7	0.43	0.42	0.010	0.010
minimum	10.3	8.8	24.6	18.3	-57.7	-19.4	-68.7	-64.7	0.20	0.19	0.002	0.002
maximum	104.6	71.4	285.8	266.2	52.6	39.9	171.1	174.6	0.86	0.76	0.027	0.021

[α] RCs ratio of grain concentration (Bq kg^{-1}) to soil concentration (Bq kg^{-1}); [β] -, not measured.

Supplementary Table S3. Agronomic traits of each variety evaluated in this study.

Variety No.	HD$^{\alpha}$	CL$^{\beta}$	SN$^{\gamma}$	GW$^{\delta}$	100GW$^{\varepsilon}$	GN$^{\zeta}$
1	211	112	7.59	13.53	4.80	37.2
2	211	107	6.47	11.09	4.50	38.1
3	212	85	3.57	4.66	4.16	31.8
4	210	119	7.58	10.98	3.56	40.6
5	201	97	5.57	11.47	3.33	59.8
6	197	73	6.24	10.66	3.45	49.0
7	199	55	7.92	12.93	3.25	50.3
8	199	84	4.65	9.90	4.07	53.3
9	196	69	3.96	5.77	3.40	42.9
10	204	84	4.11	6.29	3.11	48.7
11	206	78	5.23	11.86	3.61	64.3
12	195	61	5.71	7.92	2.98	46.8
13	205	70	4.39	7.99	3.71	47.9
14	197	78	4.83	7.31	3.25	46.8
15	198	53	6.65	10.76	3.08	53.0
16	198	81	6.49	12.15	3.37	55.6
17	201	108	7.21	14.75	4.53	45.1
18	196	51	5.00	6.86	2.83	48.7
19	196	59	6.48	10.94	3.06	54.7
20	198	66	6.65	13.02	3.57	54.7
21	197	71	4.04	7.38	3.09	59.5
22	202	91	5.42	13.52	3.56	69.5
23	205	108	6.81	11.50	3.84	43.6
24	198	68	4.77	8.22	3.48	49.9
25	197	74	5.14	6.49	3.03	41.6
26	196	67	3.70	6.77	3.27	54.8
27	197	71	5.20	9.60	3.46	53.5
28	197	52	5.83	8.41	3.08	46.3
29	199	69	5.60	9.23	3.51	48.2
30	200	62	4.15	4.82	2.81	40.0
31	203	75	6.49	16.44	3.43	74.2
32	196	71	4.04	5.99	3.35	44.3
33	202	94	5.52	12.85	3.32	69.9
34	200	64	7.41	11.52	2.85	54.3
35	197	83	4.41	6.46	3.80	40.3
36	197	76	3.82	6.93	3.40	55.8

Supplementary Table S3. (Continued).

Variety No.	HD^{α}	CL^{β}	SN^{γ}	GW^{δ}	$100GW^{\varepsilon}$	GN^{ζ}
37	198	86	5.75	9.16	3.01	53.7
38	202	85	8.63	19.51	3.42	69.2
39	194	71	7.94	13.75	3.50	49.5
40	204	81	5.57	8.62	3.03	50.3
41	202	93	6.29	12.15	3.39	56.4
42	204	83	10.16	12.73	2.68	47.8
43	203	87	7.50	9.05	2.72	44.5
44	198	78	6.25	10.09	3.29	49.1
45	209	108	10.79	15.97	4.30	34.5
46	197	74	3.81	5.19	3.26	41.3
47	205	71	4.57	10.91	3.32	72.1
48	195	75	3.50	5.89	4.26	39.4
49	198	75	4.35	8.58	3.88	51.1
50	195	61	3.73	7.19	3.71	52.1
51	199	75	2.93	5.17	3.97	44.5
52	195	66	5.31	7.49	3.38	41.6
53	198	62	4.61	9.72	3.69	55.8
54	212	89	8.40	11.42	3.94	35.7
55	195	55	3.95	5.50	3.44	40.3
56	201	49	8.30	11.52	3.72	38.0
57	196	65	7.27	13.81	4.05	46.6
58	196	63	3.92	7.23	3.69	48.8
59	203	79	4.54	7.30	4.96	32.1
60	206	80	8.71	22.13	4.12	61.3
61	196	63	2.39	3.02	3.57	35.6
62	196	56	4.80	7.92	3.19	51.5
63	196	65	3.17	6.06	3.65	52.0
64	200	67	7.04	11.26	3.51	45.6
65	197	63	7.21	13.85	3.68	52.1
66	203	59	4.83	8.46	3.34	52.8
67	206	100	6.48	10.46	3.74	43.7
68	209	107	10.45	16.02	4.42	34.9
69	210	80	8.18	14.28	4.09	42.5
70	206	71	6.81	12.82	3.52	53.5
71	201	60	4.40	6.86	3.54	44.2
72	207	91	4.96	9.93	4.06	49.2

Supplementary Table S3. (Continued).

Variety No.	HD$^{\alpha}$	CL$^{\beta}$	SN$^{\gamma}$	GW$^{\delta}$	100GW$^{\varepsilon}$	GN$^{\zeta}$
73	199	68	5.46	10.15	3.96	47.5
74	206	74	5.46	10.69	3.48	56.2
75	201	64	2.52	5.21	4.13	50.3
76	198	56	5.00	7.54	3.66	41.2
77	198	71	7.13	10.75	3.93	38.5
78	196	53	4.05	5.68	3.90	35.7
79	194	60	3.87	5.37	3.35	41.5
80	192	56	5.90	8.46	3.47	41.5
81	205	64	6.47	13.87	3.74	57.6
82	208	62	7.01	11.83	3.21	52.6
83	206	83	4.15	7.82	3.86	48.7
84	197	68	4.95	8.96	4.22	42.6
85	202	71	5.44	15.07	3.98	69.9
86	196	64	7.11	13.78	3.67	51.6
87	193	55	3.62	5.14	3.87	36.6
88	194	55	5.20	8.61	3.53	47.6
89	193	61	3.48	5.28	3.93	38.1
90	194	69	4.38	6.07	3.28	41.4
91	197	54	3.03	5.59	3.61	51.0
92	199	65	6.20	11.80	3.50	54.3
93	196	60	4.87	9.66	3.39	58.4
94	200	64	3.71	4.60	5.67	22.0
95	206	88	6.07	12.24	2.99	67.9
96	212	105	5.12	6.33	4.02	31.3
97	197	68	6.11	10.18	3.73	43.5
98	202	104	4.10	7.39	5.10	35.7
99	206	88	4.41	6.24	3.85	36.7
100	202	80	6.28	9.29	4.20	35.3
101	211	106	9.56	12.81	3.48	38.3
102	218	104	5.56	10.45	3.75	50.3
103	223	71	4.39	0.49	3.78	2.9
104	218	104	5.38	7.39	4.40	27.0
105	220	84	5.23	4.87	3.82	24.8
106	204	79	4.33	7.14	2.87	58.6
107	200	93	4.30	9.23	3.83	55.0
108	201	93	4.48	6.70	4.93	31.4

Supplementary Table S3. (Continued).

Variety No.	HD$^{\alpha}$	CL$^{\beta}$	SN$^{\gamma}$	GW$^{\delta}$	100GW$^{\varepsilon}$	GN$^{\zeta}$
109	196	64	4.30	9.21	5.40	39.4
110	205	99	6.47	-$^{\eta}$	-	-
111	196	51	4.76	5.67	2.84	42.2
112	208	102	5.10	8.69	4.26	39.7
113	211	88	4.06	6.25	4.89	31.3
114	205	110	7.56	10.47	4.19	33.0
115	213	86	4.20	5.89	4.41	31.9
116	213	92	5.08	9.18	4.58	39.4
117	208	107	7.24	13.80	4.76	40.4
118	199	84	5.56	8.09	2.77	52.5
119	212	100	7.69	13.90	3.19	56.5
120	202	99	4.60	9.79	4.55	45.3
121	207	101	4.22	6.36	4.55	31.3
122	207	89	2.22	5.22	4.10	58.6
123	217	116	3.96	6.91	4.28	42.3
124	210	123	8.46	12.12	4.34	32.6
125	206	84	4.58	11.46	4.66	53.4
126	207	77	4.77	11.52	4.68	51.7
127	216	94	5.27	8.18	3.43	45.0
128	210	77	3.45	5.62	5.27	31.2
129	206	87	2.88	5.44	3.67	40.8
130	206	75	4.42	9.01	4.34	46.7
131	206	89	4.83	8.25	4.04	42.1
132	202	81	3.43	6.17	5.44	33.1
133	204	84	3.80	3.94	6.04	17.0
134	198	76	2.91	3.58	5.05	24.2
135	201	71	1.13	1.70	5.18	24.8
136	206	88	4.40	5.35	5.54	21.6
137	208	98	3.72	6.94	5.08	37.5
138	207	90	2.72	5.79	4.47	47.8
139	208	91	4.14	5.38	4.39	29.8
140	209	86	3.75	5.26	5.56	25.7
141	204	100	3.46	6.78	5.01	39.2
142	204	83	4.43	1.57	5.85	6.5
143	205	82	2.56	5.62	5.70	37.2
144	205	82	2.23	3.88	4.97	35.4

Supplementary Table S3. (Continued).

Variety No.	HD$^{\alpha}$	CL$^{\beta}$	SN$^{\gamma}$	GW$^{\delta}$	100GW$^{\varepsilon}$	GN$^{\zeta}$
145	205	74	1.83	2.89	4.92	31.1
146	204	88	3.42	3.99	2.82	41.2
147	202	81	2.29	2.73	3.35	39.7
148	204	84	3.31	5.94	4.70	37.0
149	208	87	4.89	6.64	3.28	39.8
150	211	84	3.93	7.41	4.03	47.2
151	212	84	1.92	1.39	5.86	11.9
152	210	93	3.12	5.58	4.87	36.9
153	202	72	2.48	3.27	4.26	30.2
154	203	85	3.25	5.22	3.57	44.8
155	198	65	2.98	4.43	4.23	35.3
156	199	81	4.40	6.21	4.69	27.1
157	203	90	1.84	3.84	5.47	37.9
158	201	84	1.47	2.69	5.38	34.4
159	203	94	2.81	3.86	4.65	29.5
160	199	77	3.18	6.75	4.26	49.6
161	202	90	4.06	6.01	4.96	29.7
162	203	95	3.66	5.11	4.44	31.5
163	208	84	4.46	5.61	4.35	28.9
164	207	100	4.46	6.36	5.02	28.6
165	211	85	4.05	5.44	4.25	31.6
166	211	108	8.52	14.07	4.38	37.8
167	208	96	5.89	9.40	4.35	37.7
168	204	67	2.57	5.10	4.08	48.7
169	205	88	2.91	4.33	4.83	27.0
170	203	91	4.47	7.40	4.15	40.7
171	206	97	5.86	8.80	2.83	52.5
172	196	81	6.37	7.06	2.50	44.1
173	205	107	5.61	7.86	2.98	46.8
174	200	75	2.99	3.14	2.69	39.2
175	200	95	5.93	8.90	2.62	57.9
176	196	73	6.78	6.65	2.69	36.7
177	196	75	6.23	6.67	3.23	34.7
178	210	97	8.65	16.81	3.19	60.0
179	206	98	3.59	6.87	4.48	42.8
180	198	85	4.14	9.78	4.20	56.7

Supplementary Table S3. (Continued).

Variety No.	HD$^{\alpha}$	CL$^{\beta}$	SN$^{\gamma}$	GW$^{\delta}$	100GW$^{\varepsilon}$	GN$^{\zeta}$
181	197	83	5.29	12.10	4.13	55.3
182	205	92	3.23	5.56	4.79	36.1
183	201	85	3.38	5.89	4.63	37.5
184	206	86	4.27	5.09	5.23	22.5
185	209	70	2.00	2.09	4.31	25.3
186	205	79	2.64	5.06	5.13	37.1
187	203	74	2.53	1.65	5.72	12.3
188	201	74	3.17	4.40	3.88	35.9
189	201	76	4.20	3.75	3.72	23.8
190	203	91	4.15	6.23	3.87	38.9
191	203	91	2.99	5.44	4.40	41.5
192	200	80	3.88	5.34	4.18	33.1
193	205	114	5.33	7.80	4.76	31.1
194	208	76	3.84	4.19	3.97	27.5
195	209	89	3.89	5.83	5.43	26.3
196	203	89	4.14	5.54	5.59	24.5
197	204	85	5.54	8.87	4.83	33.6
198	208	90	4.76	7.76	5.36	30.6
199	206	95	4.62	8.18	5.22	33.8
200	209	91	4.31	5.61	4.34	29.8
201	218	106	8.48	9.28	3.14	34.8
202	209	101	6.59	9.58	4.26	34.3
203	203	74	3.36	5.17	4.43	34.9
204	205	90	3.31	5.32	4.99	31.7
205	199	54	2.25	5.90	4.74	56.2
206	211	60	3.93	6.44	3.48	48.1
207	213	77	1.22	1.49	4.18	29.2
208	216	95	3.16	5.93	5.02	42.7
209	213	94	3.63	6.88	4.12	44.7
210	210	92	4.13	10.23	4.79	51.6
211	207	87	2.55	6.49	4.14	61.2
212	208	91	1.13	2.51	4.33	49.3
213	212	87	4.52	6.53	3.71	39.6
214	202	88	6.94	14.96	3.31	65.2
215	211	87	3.18	2.83	5.21	15.4
216	212	78	1.55	2.26	4.28	34.1

Supplementary Table S3. (Continued).

Variety No.	HD[α]	CL[β]	SN[γ]	GW[δ]	100GW[ε]	GN[ζ]
217	213	76	5.22	6.21	4.97	19.9
218	197	69	4.54	4.17	3.61	25.2
219	200	56	1.14	1.48	3.85	31.3
220	202	56	3.64	2.49	2.86	23.7
221	199	69	4.46	9.50	3.65	59.6
222	200	74	4.63	6.32	4.20	32.5
223	209	83	2.22	2.86	4.06	32.5
224	212	84	7.71	11.81	3.78	40.6
225	198	60	5.60	9.83	3.92	44.9
226	198	64	2.09	3.03	3.37	42.8
227	202	78	5.80	8.02	3.65	37.6
228	204	71	3.01	7.35	3.95	61.7
229	194	57	4.64	6.84	3.28	45.0
230	189	45	3.38	3.58	3.98	26.5
231	193	52	5.75	6.93	3.45	35.0
232	195	59	5.82	10.21	3.52	49.7
233	199	82	5.72	10.03	4.05	45.2
234	195	66	5.05	8.21	2.93	54.1
average	203	80	4.81	7.88	3.99	42.1
minimum	189	45	1.13	0.49	2.50	2.9
maximum	223	123	10.79	22.13	6.04	74.2

[α] Days from sowing to heading; [β] Culm length; [γ] Spike number per plant; [δ] Grain weight per plant; [ε] 100 grain weight; [ζ] Grain number per spike; [η] -, not measured.
